# The complete mitochondrial genome of Melon thrips, *Thrips palmi* (Thripinae): Comparative analysis

**DOI:** 10.1371/journal.pone.0199404

**Published:** 2018-10-31

**Authors:** Rajasree Chakraborty, Kaomud Tyagi, Shantanu Kundu, Iftikar Rahaman, Devkant Singha, Kailash Chandra, Srinivas Patnaik, Vikas Kumar

**Affiliations:** 1 Centre for DNA Taxonomy, Molecular Systematics Division, Zoological Survey of India, New Alipore, Kolkata, West Bengal, India; 2 School of Biotechnology, Kalinga Institute of Industrial Technology (KIIT), Deemed to be University, Bhubaneswar, Odisha, India; Chinese Academy of Agricultural Sciences Institute of Plant Protection, CHINA

## Abstract

The melon thrips, *Thrips palmi* is a serious pest and vector for plant viruses on a wide range of economically important crops. DNA barcoding evidenced the presence of cryptic diversity in *T*. *palmi* and that warrants exhaustive molecular studies. Our present study is on decoding the first complete mitochondrial genome of *T*. *palmi* (15,333 bp) through next-generation sequencing (NGS). The *T*. *palmi* mt genome contains 37 genes, including 13 Protein coding genes (PCGs), two ribosomal RNA (rRNAs), 22 transfer RNA (tRNAs), and two control regions (CRs). The majority strand of *T*. *palmi* revealed 78.29% A+T content, and 21.72% G+C content with positive AT skew (0.09) and negative GC skew (-0.06). The ATN initiation codons were observed in 12 PCGs except for *cox1* which have unique start codon (TTG). The relative synonymous codon usage (RSCU) analysis revealed Phe, Leu, Ile, Tyr, Asn, Lys and Met were the most frequently used amino acids in all PCGs. The codon (CGG) which is assigned to Arginine in most insects but absent in *T*. *palmi*. The Ka/Ks ratio ranges from 0.078 in *cox1* to 0.913 in *atp8*. We observed the typical cloverleaf secondary structure in most of the tRNA genes with a few exceptions; absence of DHU stem and loop in *trnV* and *trnS*, absence of DHU loop in *trnE*, lack of T-arm and loop in *trnN*. The *T*. *palmi* gene order (GO) was compared with ancestral GO and observed an extensive gene arrangement in PCGs, tRNAs and rRNAs. The *cox2* gene was separated from the gene block ‘*cox2*-*trnL2*’ in *T*. *palmi* as compared with the other thrips mt genomes, including ancestor GO. Further, the *nad1*, *trnQ*, *trnC*, *trnL1*, *trnV*, *trnF*, *rrnS*, and *rrnL* were inversely transpositioned in *T*. *palmi* GO. The gene blocks ‘*trnQ*-*trnS2*-*trnD*’ and ‘*trnN*-*trnE*-*trnS1*-*trnL1*’ seems to be genus specific. The *T*. *palmi* mt genome contained 24 intergenic spacer regions and 12 overlapping regions. The 62 bp of CR2 shows the similarity with CR1 indicating a possible duplication. The occurrence of multiple CRs in thrips mt genomes seems to be a derived trait which needs further investigation. Although, the study depicted extensive gene rearrangements in *T*. *palmi* mt genome, but the negative GC skew reflects only strand asymmetry. Both the ML and BI phylogenetic trees revealed the close relationships of *Thrips* with *Scirtothrips* as compared to *Frankliniella*. Thus, more mt genomes of the diverse thrips species are required to understand the in-depth phylogenetic and evolutionary relationships.

## Introduction

The members of insect order Thysanoptera (commonly known as thrips) are usually tiny, fringe winged and are classified into nine families within two suborders [1]. The family Thripidae is the most diverse family and further divided into four subfamilies (Dendrothripinae, Panchaetothripinae, Sericothripinae and Thripinae). Thrips are widely distributed throughout the world and known by 6154 species, of which 739 species are reported from India [2]. Thrips are one of the major sucking pests and sole transmitters of Tospoviruses (Family Bunyaviridae) on a wide number of agricultural and horticultural crops [3]. Fifteen species are reported as vectors of tospoviruses, of which six species are known from India (*Ceratothripoides claratris*, *Frankliniella occidentalis*, *Frankliniella schultzei*, *Scirtothrips dorsalis*, *Thrips palmi* and *Thrips tabaci*) [4–5]. Due to their economic importance, the accurate identification of these species is the basic necessity; for the study of their disease transmission efficiency in crops, and implementation of the effective management strategies. The morphological identification is time consuming and challenging because of their cryptic behaviour and overlapping geographical distributions. Therefore, modern molecular tools such as DNA barcoding have been applied for thrips identification and phylogenetic studies [6–8].

*Thrips palmi* is commonly known as melon thrips, one of the major pest on agricultural crops and has been reported as vector for four tospoviruses; Calla lily chlorotic spot virus (CCSV), Groundnut bud necrosis virus (GBNV), Melon yellow spot virus (MYSV), Watermelon silver mottle virus (WSMV) [4]. The species is widely distributed and highly polyphagous and is often confused with the *Thrips flavus* and *Thrips alatus* [9]. Recent DNA barcoding studies on thrips, revealed four molecular operations taxonomic units (MOTUs) in *T*. *palmi* (*T*. *palmi* Ia1, *T*. *palmi* IIa1, *T*. *palmi* Ib1, and *T*. *palmi* Ib2) representing multiple cryptic species [8]. Considering these hitches, more and in-depth molecular data on this species is required to understand the cryptic speciation and evolutionary affiliations.

Mitochondrial genome (mt genome) data have been widely used for phylogenetic, evolutionary studies, and population genetics in insects [10–11]. The mt genomes of insects were usually represented by 37 genes, including 13 PCGs, large and small ribosomal RNA genes (*rrnL* and *rrnS*), 22 transfer RNA genes (tRNAs), variable number of control regions (CRs) and chromosomes [12–14]. However, the availability of thrips mt genomes is limited in global database and six mt genomes of five species (*Anaphothrips obscurus*, *Frankliniella intonsa*, *Frankliniella occidentalis*, *Scirtothrips dorsalis* and *Thrips imaginis*) are available in the GenBank [15–19].

In the present study, we sequenced and characterized the complete mt genomes of the melon thrips, *T*. *palmi* under the family Thripidae using the next generation sequencing (NGS) technology and comparative analysis with other thrips mt genomes. The comparisons were based on gene arrangements, nucleotide composition, codon usage, evolutionary rates, and strand asymmetry etc. Further, to infer the phylogenetic relationships, 13 PCGs of *T*. *palmi* and other six thrips species mt genomes were analyzed using maximum likelihood (ML) and Bayesian inference (BI). This study will provide a better understanding of comparative mitochondrial genomics *T*. *palmi* with thysanopterans and other insects.

## Materials and methods

### Ethics statement

There is no need of specific permission for the collection and usage of the insects in this study because these insects are common pests of crops. Neither endangered nor protected species were involved in the study.

### Sample collection, and DNA extraction

The specimens of *T*. *palmi* were collected from the Odisha state of India from Eggplant (*Solanum melongena*). The specimens were morphologically identified by the second author (K.T) with the available taxonomic keys [9], and preserved in absolute ethyl alcohol at −80°C in Centre for DNA Taxonomy, Molecular Systematics Division, Zoological Survey of India, Kolkata. The DNeasy DNA Extraction kit (Qiagen, Valencia, CA) was used to extract the genomic DNA and the concentration was measured on a quantified by Qubit fluorometer (Thermo Fisher Scientific, MA, USA) using a dsDNA high-sensitivity kit with the standard protocol.

### Mitochondrial genome sequencing and assembly

The complete mt genome sequencing, assembly and annotation were carried out at the Genotypic Technology Pvt. Ltd. Bangalore, India (http://www.genotypic.co.in/). Whole genome sequencing (WGS) library was prepared with Illumina-compatible NEXTflex Rapid DNA sequencing kit (BIOO Scientific, Austin, Texas, U.S.A.). The DNA was sheared using a Covaris S2 sonicator (Covaris, Woburn, Massachusetts, USA) to generate approximate fragment size distribution of 200 bp to 600 bp. The fragment size distribution was checked on Agilent TapeStation and subsequently purified using Highprep magnetic beads (Magbio). Purified fragments were end-repaired, adenylated and ligated to Illumina multiplex barcode adaptors. Adapter-ligated DNA was purified using Highprep beads and the resulted fragments were amplified for eight cycles of PCR using Illumina-compatible primers. The final PCR product was purified, followed by library quality control check. Illumina-compatible sequencing library was quantified by Qubit fluorometer (Thermo Fisher Scientific, MA, USA) and its fragment size distribution were analyzed by Agilent 2200 Tapestation and Agilent Bioanalyzer. Libraries with adapter contamination were pooled and cleaned up with Highprep magnetic beads (Magbio) and then sequenced on Nextseq 500 150X2 chemistry. Raw Sequences were trimmed and filtered using NGS Toolkit. The adapter contamination and low-quality reads with base N’s or more than 70% of the bases with a quality score <20 also had been removed. The acquired high quality ~18 million reads were screened out using the Burrows-Wheeler Alignment (BWA) tool [20]. Out of 18 million reads, 0.10% (~1.8 million) of the reads got aligned, then assembled with SPAdes 3.9.0 [21], using default parameters considering *T*. *imaginis* mitochondrial genome (AF335993) as reference contig. The aligned reads were considered for the denovo mitochondrial genome of *T*. *palmi*.

### Genome annotation, visualization, and comparative analysis

The assembled mt genome was annotated by using MITOS web-server (http://mitos.bioinf.uni-leipzig.de/index.py) to estimate the position of PCGs, tRNAs, rRNAs and their secondary structures. The boundaries of PCGs and rRNAs was confirmed manually by nucleotide-nucleotide BLAST (BLASTn), protein-protein BLAST (BLASTp), and open reading frame finder (ORF finder) in the National Center for Biotechnology Information (NCBI) (https://www.ncbi.nlm.nih.gov/orffinder/). The PCGs were translated into putative proteins using the invertebrate mitochondrial DNA genetic code. The ClustalX program was used to assign the initiation and termination codons in comparison with other thrips reference sequences [22]. MEGAX was used for alignment of the homologous sequences of *T*. *palmi* with other thrips species [23]. The complete annotated *T*. *palmi* mt genome was prepared using the Sequin submission tool (http://www.ncbi.nlm.nih.gov/Sequin/), for acquiring the accession number from GenBank database. The circular representation of *T*. *palmi* mt genome was drawn by CGView online server (http://stothard.afns.ualberta.ca/cgview_server/) with default parameters [24]. The assembled *T*. *palmi* mt genome was compared with the other thrips mt genomes to calculate the nucleotide composition, Relative Synonymous Codon Usage (RSCU), AT- GC skew, non-synonymous (Ka) and synonymous (Ks) substitutions, gene rearrangements, secondary structure of tRNAs and CRs etc. The nucleotide composition and RSCU were determined using MEGAX [23]. The following formula was used to calculate the skew: AT skew = (A−T) / (A+T) and GC skew = (G−C) / (G+C) [25]. The sequence substitution saturation analysis of PCGs was calculated by DAMBE5 software [26]. The overlapping and intergenic spacer regions of thrips mt genome genes were compared in terms of length and locations. Further the homology of CRs in *T*. *palmi* and other Thrips mt genomes were determined through sequence alignment using Clustal Omega [22]. The nucleotide sequences of each PCG were aligned based on the amino acid sequences in TranslatorX [27] with MAFFT algorithm using GBlocks parameters. The ratios of non-synonymous substitutions (Ka) and synonymous (Ks) substitutions were estimated in DnaSP6.0 [28]. The secondary structures of tRNAs were anticipated by the MITOS web server (http://mitos.bioinf.uni-leipzig.de) and confirmed by the tRNAscan-SE (http://lowelab.ucsc.edu/tRNAscan-SE/) [29] and ARWEN 1.2 [30]. The RNAstructure version 6.0.1 was used to predict the secondary structure of CRs [31]. The gene arrangements, pairwise comparisons of gene orders, and evolutionary pathways of *T*. *palmi* mt genome were evaluated by CREx web tool (Common Interval Rearrangement Explorer) [32]. This web-based program uses transpositions, reverse transpositions, reversals and tandem-duplication-random-loss (TDRL) events to find the parsimonious rearrangement events in a phylogenetic hypothesis. CREx analysis based on common intervals was implemented to determine the gene arrangement scenarios between the *T*. *palmi* gene order (GO) with the ancestral *A*. *bakeri* (Hemiptera) and other thrips species.

### Phylogenetic analysis

Six complete mt genomes of five thrips species were retrieved from GenBank on April 1^st^, 2018 for phylogenetic inference (S1 Table). The *A*. *bakeri* mt genome was used in the dataset as an out-group [33]. The PCGs were individually aligned with the TranslatorX online platform using the MAFFT algorithm [27] with the GBlocks parameters and default settings. The dataset of all PCGs was concatenated using SequenceMatrix v1.7.845 to form 9696 bp dataset [34]. The optimal substitution model was estimated by jModelTest [35]. The dataset was analyzed using maximum likelihood (ML) implemented in RaxML, and Bayesian inference (BI) implemented in MrBayes 3.2 [36]. For likelihood analysis, the bootstrap analysis of 1,000 replicates was performed in RaxML [37] with GTR+G+I as a best fit model. For BI analysis, two simultaneous runs of 12 million generations were conducted for the dataset using GTR+G+I model and trees were sampled in every 1000 generations, with the first 25% discarded as burn-in. The BI analysis was stopped after reaching the stationary phase and average standard deviation of split frequencies below 0.01. The phylogenetic tree was visualized and edited using FigTree v1.4.2 (http://tree.bio.ed.ac.uk/software/figtree/) [38].

## Results and discussion

### Genome structure and nucleotide composition

**Fig 1:**
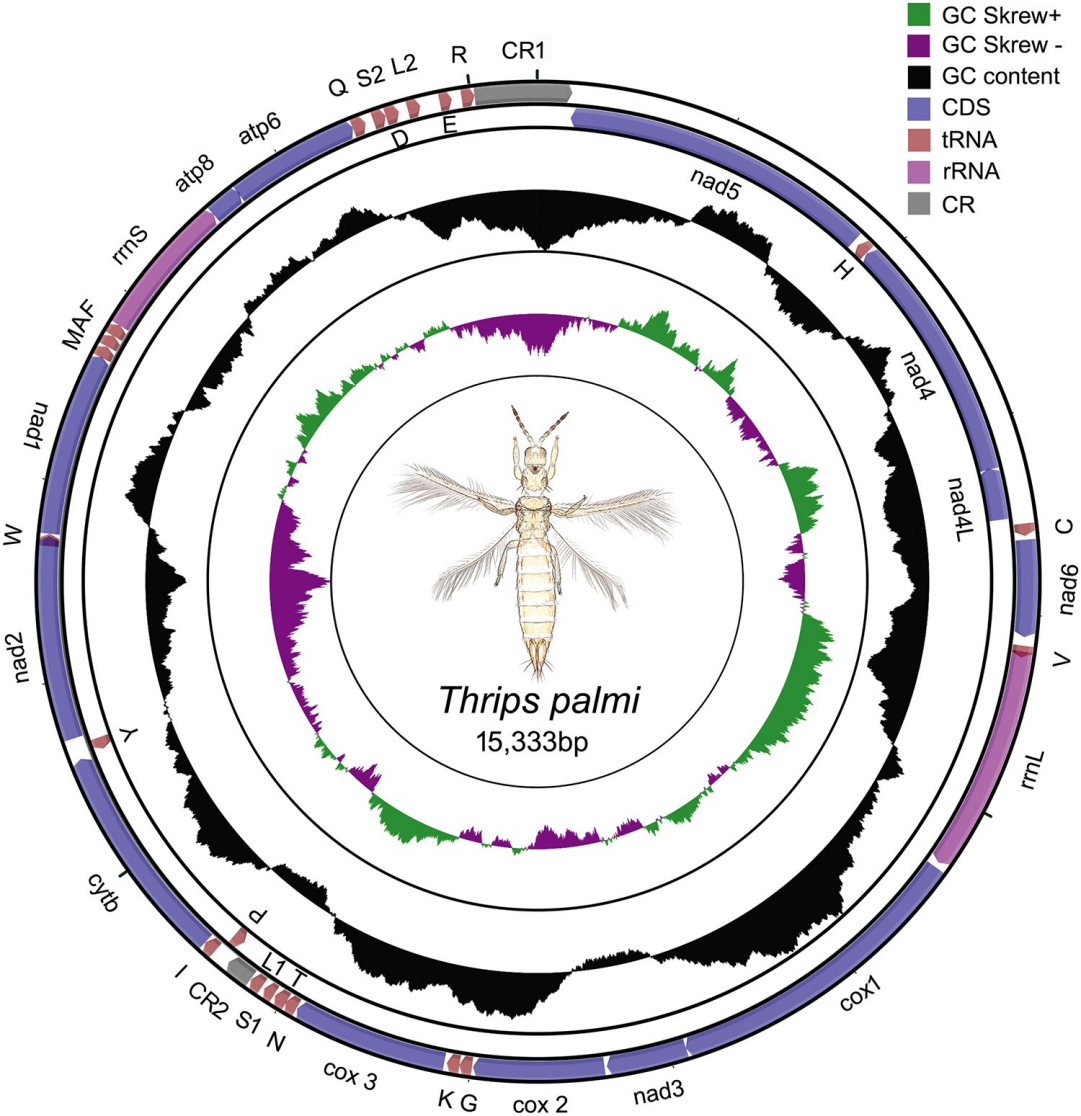
The circular representation of the complete mitochondrial genome of *T*. *palmi*. Direction of gene transcription is indicated by arrows in entire complete genome. PCGs are shown as purple arrows, rRNA genes as pink arrows, tRNA genes as peach color arrows and CR regions as gray rectangles. The GC content is plotted using a black sliding window, as the deviation from the average GC content of the entire sequence. The GC-skew is plotted using a colored sliding window (green and orchid color), as the deviation from the average GC skew of the entire sequence. The figure was drawn using the CGView online server (http://stothard.afns.ualberta.ca/cgview_server/) with default parameters. The species photograph was taken by the second author (KT) using Leica Microscope DM1000 with Leica software application suite (LAS EZ) and edited manually in Adobe Photoshop CS 8.0.

The complete mt genome of *T*. *palmi* (GenBank accession no. MH253898) was 15,333 base pairs (bp) in length. This is the second largest mt genome in size among all previously generated mt genomes of insect order Thysanoptera (S1 Table). The *T*. *palmi* mt genome was characterized by 37 genes, including 13 PCGs, large and small ribosomal RNA genes (*rrnL* and *rrnS*), 22 transfer RNA genes (tRNAs) and two control regions (CRs) (Fig 1). Among all genes, 31 genes were detected on the majority (light) strand and six genes on the minority (heavy) strand (Table 1). The nucleotide composition of *T*. *palmi* mt genome was 78.29% A+T content (42.71% A + 35.58% T) and 21.72% G+C content (10.14% G + 11.58% C) (Table 2). The A+T content was higher in control regions (85.81%) followed by tRNAs (80.26%), rRNAs (80.21%) and PCGs (77.43%). The sequence similarity searches of *T*. *palmi* mt genome showed the highest similarity (81%) with *T*. *imaginis* followed by *F*. *occidentalis* (74%), *F*. *intonsa* (73%), *S*. *dorsalis* SA1 (72%), *S*. *dorsalis* EA1 (71%) and *A*. *obscurus* (72%) in GenBank.

**Table 1:** List of annotated mitochondrial genes of *T*. *palmi* and its characteristic features. The PCGs and rRNA genes are represented by standard nomenclature, tRNAs are represented as *trn* followed by the IUPAC-IUB single letter amino acid codes. (+) values in strand represent as heavy (H) and (-) values represent as light (L). IGN represents (+) values as intergenic nucleotides and (-) values as overlapping regions. CR represents the control region.

Gene	Strand	Location	Size (bp)	Anticodon	Start Codon	Stop Codon	IGN
*nad5*		176–1828	1653	.	ATT	TAA	29
*trnH*	-	1858–1919	62	CAC	.	.	2
*nad4*	-	1922–3241	1320	.	ATT	TAA	-7
*nad4l*	-	3235–3510	276	.	ATG	TAG	34
*trnC*	+	3545–3606	62	UGC	.	.	19
*nad6*	+	3626–4111	486	.	ATT	TAA	43
*trnV*	+	4155–4214	60	GUA	.	.	0
*rrnL*	+	4215–5325	1111	.	.	.	27
*cox1*	+	5353–6915	1563	.	TTG	TAA	-1
*nad3*	+	6915–7319	405	.	ATG	TAA	12
*cox2*	+	7332–7988	657	.	ATA	TAA	5
*trnG*	+	7994–8057	64	GGA	.	.	-1
*trnK*	+	8057–8120	64	AAA	.	.	13
*cox3*	+	8134–8916	783	.	ATA	TAA	4
*trnN*	+	8921–8983	63	AAC	.	.	-3
*trnT*	+	8981–9044	64	ACA	.	.	7
*trnS1*	+	9052–9108	57	AGA	.	.	15
*trnL1*	+	9124–9188	65	CUA	.	.	3
CR2	+	9192–9329	138	.	.	.	1
*trnP*	-	9331–9394	64	CCA	.	.	23
*trnI*	+	9418–9481	64	AUC	.	.	1
*cytb*	+	9483–10595	1113	.	ATA	TAA	8
*trnY*	-	10604–10666	63	UAC	.	.	37
*nad2*	+	10704–11726	1023	.	ATA	TAA	-50
*trnW*	+	11677–11738	62	UGA	.	.	0
*nad1*	+	11739–12662	924	.	ATA	TAA	-4
*trnM*	+	12659–12719	61	AUG	.	.	1
*trnA*	+	12721–12782	62	GCA	.	.	-1
*trnF*	+	12782–12844	63	UUC	.	.	-2
*rrnS*	+	12843–13570	728	.	.	.	-1
*atp8*	+	13570–13738	169	.	ATA	T(TT)	-7
*atp6*	+	13732–14391	660	.	ATT	TAA	-1
*trnQ*	+	14391–14458	68	CAA	.	.	41
*trnS2*	+	14500–14564	65	UCA	.	.	1
*trnD*	+	14566–14633	68	GAC	.	.	46
*trnL2*	+	14680–14744	65	CUA	.	.	99
*trnE*	+	14844–14905	62	GAA	.	.	49
*trnR*	+	14955–15019	65	CGA	.	.	0
CR1	+	15020–15333+175	489	.	.	.	.

**Table 2:** Nucleotide composition and skewness in different Thysanoptera mt genomes considered for comparative analysis.

Species	Size(bp)	A%	G%	T%	C%	GC%	AT%	AT skew	GC skew
**Whole mtgenome**									
*T*. *palmi*	15,333	42.71	10.14	35.58	11.58	21.72	78.28	0.09	-0.07
*T*. *imaginis*	15,407	43.85	10.47	32.72	12.96	23.43	76.57	0.15	-0.11
*F*. *intonsa*	15,215	41.24	11.06	34.68	13.01	24.07	75.93	0.09	-0.08
*F*. *occidentalis*	14,889	40.98	11.35	36.62	11.06	22.41	77.59	0.06	0.01
*S*. *dorsalis* EA1	15,343	39.12	11.61	36.62	12.64	24.26	75.74	0.03	-0.04
*S*. *dorsalis* SA1	15,204	39.83	11.18	37.56	11.42	22.60	77.40	0.03	-0.01
*A*. *obscurus*	14,890	38.38	11.27	39.75	10.60	21.87	78.13	-0.02	0.03
**PCG**									
*T*. *palmi*	11,030	41.64	10.52	35.32	12.52	23.04	76.96	0.08	-0.09
*T*. *imaginis*	10,922	42.75	10.15	32.89	14.21	24.36	75.64	0.13	-0.17
*F*. *intonsa*	11,009	39.95	11.39	34.58	14.08	25.47	74.53	0.07	-0.11
*F*. *occidentalis*	10,852	39.82	11.62	36.72	11.84	23.46	76.54	0.04	-0.01
*S*. *dorsalis* EA1	10,954	38.06	11.92	36.53	13.48	25.41	74.59	0.02	-0.06
*S*. *dorsalis* SA1	10,973	38.94	11.36	37.67	12.03	23.38	76.62	0.02	-0.03
*A*. *obscurus*	11,167	37.36	11.46	39.93	11.25	22.71	77.29	-0.03	0.01
**tRNA**									
*T*. *palmi*	1,393	42.21	10.12	38.05	9.62	19.74	80.26	0.05	0.03
*T*. *imaginis*	1,492	43.83	9.45	36.66	10.05	19.50	80.50	0.09	-0.03
*F*. *intonsa*	1,392	43.53	10.70	35.78	9.99	20.69	79.31	0.10	0.03
*F*. *occidentalis*	1,380	42.39	10.58	37.39	9.64	20.22	79.78	0.06	0.05
*S*. *dorsalis* EA1	1,426	40.53	11.01	37.52	10.94	21.95	78.05	0.04	0.00
*S*. *dorsalis* SA1	1,429	41.36	10.43	38.21	10.01	20.43	79.57	0.04	0.02
*A*. *obscurus*	1,430	39.79	10.63	39.86	9.72	20.35	79.65	0.00	0.04
**rRNA**									
*T*. *palmi*	1839	47.36	10.82	32.68	9.14	19.96	80.04	0.18	0.08
*T*. *imaginis*	1,876	47.65	10.77	32.14	9.43	20.20	79.80	0.19	0.07
*F*. *intonsa*	1,699	47.15	11.30	32.02	9.54	20.84	79.16	0.19	0.08
*F*. *occidentalis*	1,848	45.94	12.18	33.93	7.95	20.13	79.87	0.15	0.21
*S*. *dorsalis* EA1	1,775	43.21	11.89	34.99	9.92	21.80	78.20	0.11	0.09
*S*. *dorsalis* SA1	1,777	45.36	11.65	34.44	8.55	20.20	79.80	0.14	0.15
*A*. *obscurus*	1,812	43.16	11.70	36.59	8.55	20.25	79.75	0.08	0.16
**Control region**									
*T*. *palmi*	627	44.82	4.63	40.99	9.57	14.19	85.81	0.04	-0.35
*T*. *imaginis*	900	47.56	16.67	25.22	10.56	27.22	72.78	0.31	0.22
*F*. *intonsa*	942	41.72	7.86	38.22	12.21	20.06	79.94	0.04	-0.22
*F*. *occidentalis*	595	40.34	7.90	43.70	8.07	15.97	84.03	-0.04	-0.01
*S*. *dorsalis* EA1	1,775	43.21	11.89	34.99	9.92	21.80	78.20	0.11	0.09
*S*. *dorsalis* SA1	767	35.33	9.26	43.55	11.86	21.12	78.88	-0.10	-0.12
*A*. *obscurus*	145	25.52	8.97	62.76	2.76	11.72	88.28	-0.42	0.53

### Protein coding genes and Relative Synonymous Codon Usage

The *T*. *palmi* mt genome was represented by 13 PCGs (*atp6*, *atp8*, *cox1*, *cox2*, *cox3*, *cytb*, *nad1*, *nad2*, *nad3*, *nad4*, *nad4L*, *nad5*, and *nad6*). The ATN initiation codons (six with ATA, four with ATT and two with ATG) was observed in 12 PCGs except *cox1* (TTG). The TTG start codon for *cox1* is unique in *T*. *palmi* as ATN start codon is observed for *cox1* in all other thrips mt genomes (S2 Table). The PCGs were terminated with TAA stop codon except *nad4L* (TAG) and *atp8* with an incomplete stop codon. The incomplete termination codons are common in other insect mt genomes which are assumed to be recovered by the post transcriptional polyadenylation [39–41]. The average A+T content of *T*. *palmi* PCGs was 77.43% with the highest of 84.06% in *nad4L* gene. However, the average G+C content of *T*. *palmi* PCGs was 22.57% with the highest of 27.9% in *cox1* gene. The nucleotide composition in all *T*. *plami* PCGs showed 41.64% Adenine (A), 35.32% Thiamine (T), 10.52% Guanine (G), and 12.52% Cytosine (C) (S1 Fig, S3 Table).

Further, the comparative analysis of PCGs in all seven mt genomes of six thrips species showed that the adenine (A) and thiamine (T) composition was higher than guanine (G) and cytosine (C) (Table 2). The RSCU data analysis of 3,676 codons in 13 PCGs of *T*. *palmi* revealed that Phenylalanine (Phe), Leucine (Leu), Isoleucine (Ile), Tyrosine (Tyr), Asparagine (Asn), Lysine (Lys), and Methionine (Met) were the most frequently used amino acids (S2 Fig, S4 Table). Comparative RSCU analysis of seven thrips mt genomes showed that Phe, Leu, Ile, Tyr, Asn, Lys and Met were most frequent amino acids with TTT (Phe), TTA (Leu), ATT (Ile), TAT (Tyr), AAT (Asn) and AAA (Lys), ATA (Met) were the most frequently used codons. In contrast, almost all the frequently used codons were ended with A/T, which may lead to the A and T bias in thrips species mt genomes. The codon CGG (Arg) and GCG (Ala) were absent in *T*. *palmi* and *S*. *dorsalis* SA1 respectively, while these codons were present in other thrips mt genomes. Both of the missing codons were preferred to end with ‘G’ in the third codon position (S3 Fig). Sequence saturation analysis of PCGs of the thrips mt genomes exhibited an increasing rate of transitions and transversions ratio along with the divergence value (S4 Fig, S5 Table).

### Non-synonymous and synonymous substitutions

**Fig 2:**
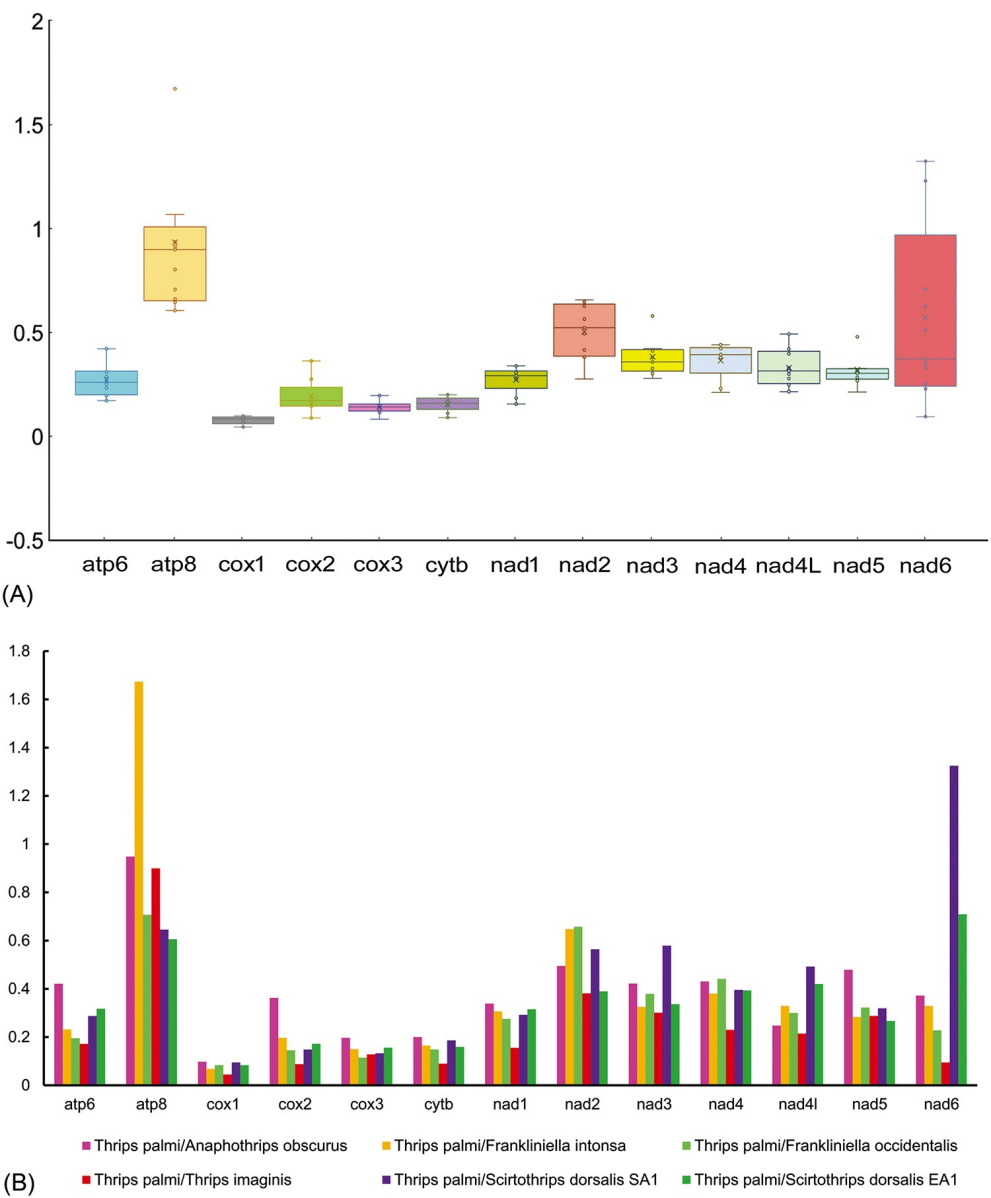
(A) Ratio estimation, box plot for pairwise divergence of Ka/Ks ratio for each one of the mitochondrial PCGs. (B) Evolutionary rates (Ka/Ks) of individuals PCGs of *T*. *palmi* with other thrips species.

The non-synonymous and synonymous substitutions (Ka/Ks) ratio is an indicator for investigating the selective pressure and evolutionary relations of the homogenous or heterogeneous species [11]. It was reported that, (i) the Ka/Ks>1 for positive selection, (ii) Ka/Ks = 1 for neutral mutation, and (iii) Ka/Ks<1 for negative selection [42–44]. The Ka/Ks ratio ranges from 0.078±0.02 in *cox1* to 0.913±0.40 in *atp8* gene and the resulted following order: *cox1*<*cox3*<*cytb*<*cox2*<*atp6*<*nad1*<*nad5*<*nad4L*<*nad4*<*nad3*<*nad6*<*nad2*<*atp8*. This result indicated that the 13 PCGs of all thrips mt genomes including *T*. *palmi* were evolving under purifying selection (Fig 2A). Comparative analysis of the Ka/Ks ratio among 13 PCGs of thrips species showed that *atp8* (1.7) and *nad6* (1.3) genes are evolving under positive/relaxed selection with reference to *F*. *intonsa* and *S*. *dorsalis* SA1 respectively (Fig 2B). Further, the Ka/Ks was <1 for the remaining PCGs of *T*. *palmi* with reference to other thrips species, suggested that the mutations were replaced by synonymous substitutions. The lowest Ka/Ks ratio was observed in *cox1* gene representing less variations in amino acids and hence had been evidenced as a potential molecular marker for thrips species identification and phylogenetic analysis [6–8].

### Ribosomal RNA and transfer RNA

The *T*. *palmi* mt genome comprises two rRNA genes as observed in other insect mt genomes. The large ribosomal gene (*rrnL*) was 1111 bp long, and located between *trnV* and *cox1*. Further, the small ribosomal gene (*rrnS*) was 728 bp long, and located between *trnF* and *atp8* gene. The A+T content of two rRNA was 80.04% in *T*. *palmi* which is the highest in comparison with other thrips mt genomes. Both AT skew (0.18) and GC skew (0.08) of *T*. *palmi* were positive, that is also similar to other previously sequenced thrips mt genomes. The locations of *rrnL* and *rrnS* were upstream of *cox1* and *atp8* gene, the arrangements seem to be conserved in insect order Thysanoptera. The *T*. *palmi* mt genome contained 22 tRNAs (ranging from 57 to 68 bp in length) with a total length of 1,393 bp. Nineteen tRNA genes were coded by the majority strand and three (*trnY*, *trnP* and *trnH*) by the minority strand. The A+T content of tRNAs was 85.81% with positive AT skew (0.05) and GC skew (0.3) (Table 2). Most of the tRNA showed the typical cloverleaf secondary structure; absence of DHU stem and loop was observed in *trnV* and *trnS;* absence of DHU loop in *trnE*; lack of TΨC loop in *trnN*; variation in TΨC arm and loop in other tRNAs (S5 Fig). The absence of DHU stem and loop in *trnV* was consistent in all the thrips mt genomes. The mismatched base pairs (G-U wobble pairs) were observed in seven tRNAs of *T*. *palmi* mt genome. These wobble mismatches were observed in *trnL2* and *trnL1* (in DHU arm), *trnA*, *trnS1*, and *trnL2* (in acceptor arm), *trnS2* and *trnT* (in TΨC arm).

### Overlapping and intergenic spacer regions

The *T*. *palmi* mt genome had 24 intergenic spacer regions with a total of 520 bp, varying from 1 to 99 bp in length. There are 14 major intergenic spacers of >10 bp in length were observed (Table 1). The comparative analysis depicted highest intergenic spacer region in *T*. *palmi* in comparison with other thrips species; 17 intergenic spacer of 217 bp in *T*. *imaginis*, 14 intergenic spacer of 172 bp in *F*. *intonsa*, 14 intergenic spacer of 217 bp in *F*. *occidentalis*, 13 intergenic spacer of 436 bp in *S*. *dorsalis* EA1, 13 intergenic spacer of 342 bp, 19 intergenic spacer of 309 bp in *A*. *obscurus*. The longest intergenic spacer (99 bp) was observed between the *trnL2* and *trnE* gene in *T*. *palmi*. However, the shortest intergenic spacer (1 bp) was observed in four positions: *trnP* and CR2, *cytb* and *trnI*, *trnA* and *trnM*, *trnD* and *trnS2*. Further, the comparative studies showed that the longest intergenic spacer 150 bp were between *trnH* and *nad4* in *S*. *dorsalis* EA1. The *T*. *palmi* mt genome contained 11 overlapping regions with a total length of 78 bp. The comparative analysis showed that, the highest (15 overlapping regions of 86 bp) were observed in *S*. *dorsalis* SA1. The smallest overlapping region (1 bp) was observed in five positions in *T*. *palmi*: *nad3* and *cox1*, *trnK* and *trnG*, *trnF* and *trnA*, *atp8* and *rrnS*, *trnQ* and *atp6*. The largest overlapping region (50 bp) was observed between *nad2* and *trnW* in *T*. *palmi*. However, the largest overlapping region (66 bp) was observed between *rrnL* and *trnS2* in *F*. *occidentalis* in comparative studies (S6 Table).

### Control regions

**Fig 3:**
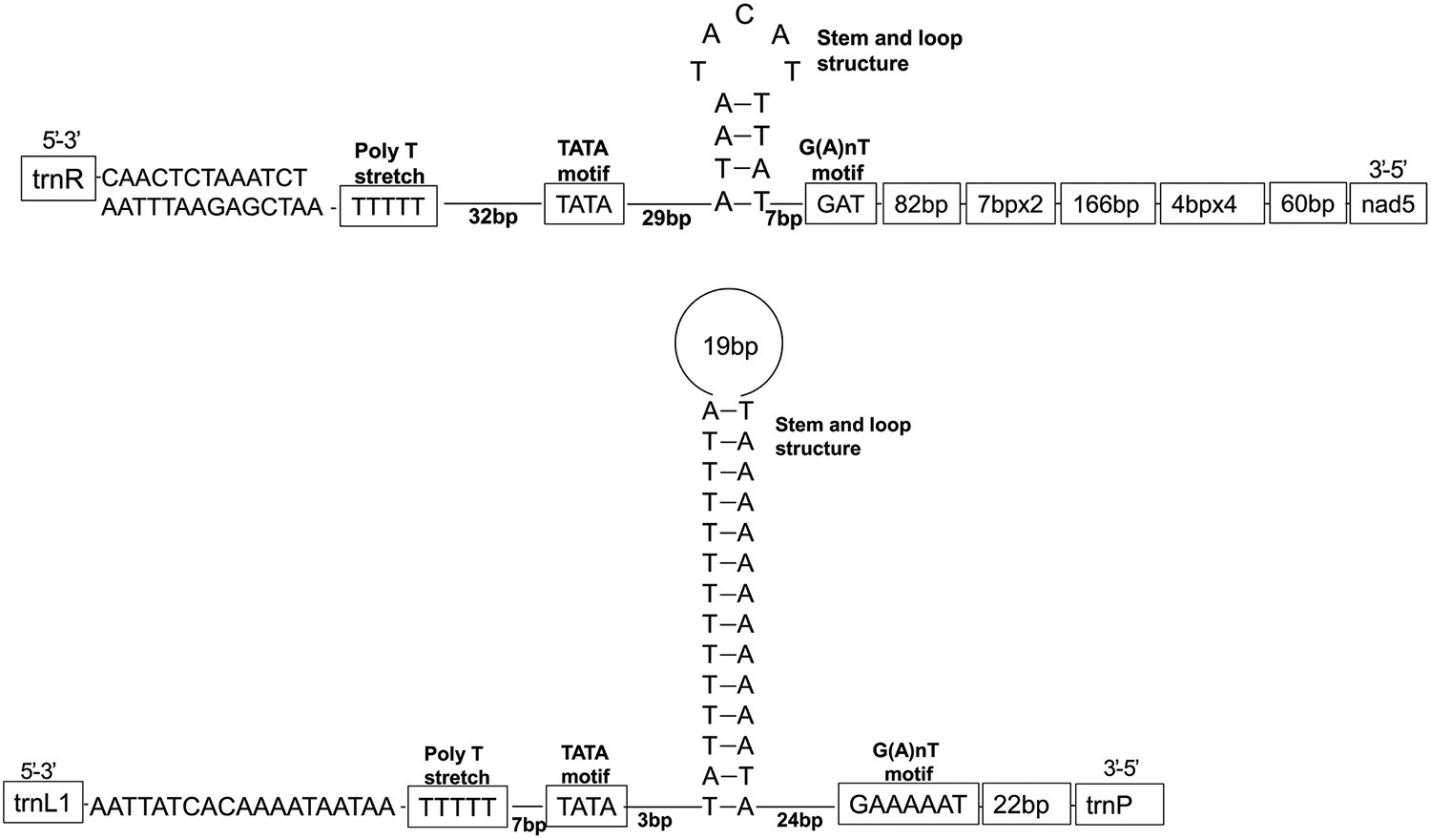
Predicted structural elements for control regions (CRs) of *T*. *palmi*.

The CRs is usually characterized by five conserved elements, such as (i) a polyT stretch at the 5′ end; (ii) a [TA(A)]n-like stretch (iii) a stem and loop structure (iv) a TATA motif and a G(A)nT motif flanking the stem and loop structure (v) a G+A-rich sequence downstream of the stem and loop structure [45–47]. Both CRs of *T*. *palmi* were detected with four conserved elements except G+A-rich sequence (Fig 3). The control region (CR) in mt genomes is most crucial region, which regulates transcription and replication [48]. Duplicate CRs has been documented in many insect species, including thrips [15–18]. The study resulted in the duplication of CRs in *T*. *palmi* mt genome; 489 bp CR1 lied between *trnR* and *nad5*, 138 bp CR2 between the *trnL1* and *trnP*. Further, we observed 62 bp sequence similarity of CR2 with CR1 indicating a possible duplication. The total length of CR1 and CR2 was 627 bp, which was higher than *A*. *obscurus* (145 bp) and *F*. *occidentalis* (595bp). *T*. *palmi* had two CRs, similar to *T*. *imaginis*, and *S*. *dorsalis* EA1. The presence of multiple CRs in thrips species that are serious pests and vectors for tospoviruses, seems to be a genomic apomorphy over other common thrips species and the ancestral species. However, more thrips mt genomes are needed to be studied to confirm, whether the duplication of CR in thrips species is linked with the association of tospoviruses.

### Gene arrangement

**Fig 4:**
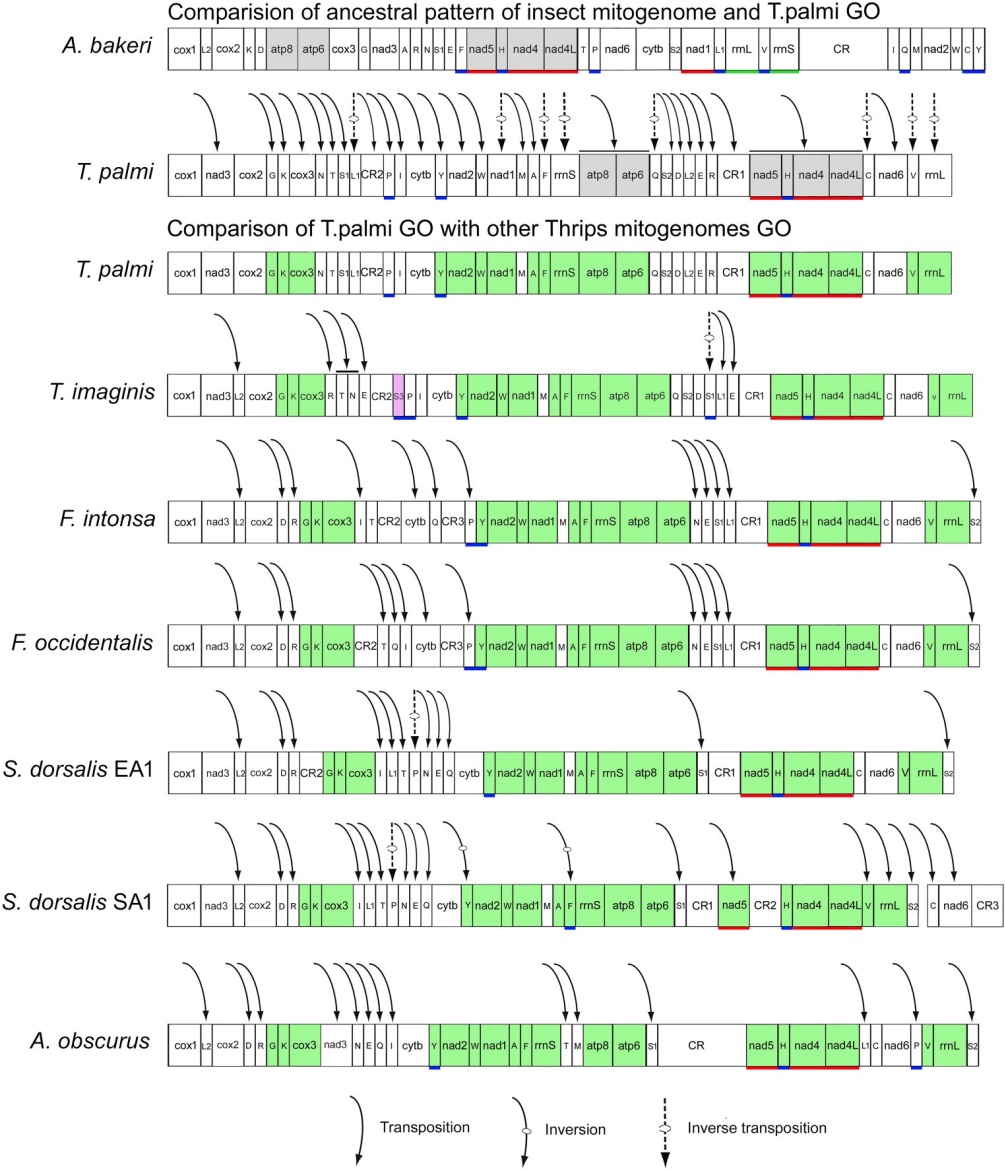
Linearized view of complete mitochondrial genome organization and gene rearrangement, transposition, inversion, and inverse transposition in *T*. *palmi* compared with the ancestral type of the insect (*A*. *bakeri*). The gray color blocks show the conserve gene blocks of *T*. *palmi* and *A*. *bakeri*. The green color blocks show the conserve gene blocks of *T*. *palmi* and other Thysanoptera species. The pink color block shows the extra tRNA present in *T*. *imaginis*. Gene blocks with underline shows the position of genes in minority strand (red for PCGs, green for rRNAs, and blue for tRNAs). Different shapes of arrows are used for showing transposition, inversion, and inverse transposition. Gene nomenclature: *atp6* and *atp8*; ATP synthase subunits 6 and 8; *cytb*: cytochrome b; *cox1*–*3*: cytochrome c oxidase subunits 1–3; *nad1*–*6* and *nad4L*: NADH dehydrogenase subunits *1*–*6* and *4L*; *rrnS* and *rrnL*: small and large subunit ribosomal RNA (rRNA) genes; Transfer RNA genes are denoted by a one-letter symbol according to the IPUCIUB single-letter amino acid codes. CR indicates the control region.

The gene arrangements can be categorized by following aspects, (i) transpositions, (ii) inversions, (iii) inverse transpositions, and (iv) the tandem duplication random loss operation (TDRL) [49–51]. The gene arrangement of *T*. *palmi* was evaluated by comparing the common intervals with *A*.*bakeri* GO, which was assumed to be the putative ancestor of hexapods [19, 33]. The CREx identified seven operations in the evolution of gene arrangement in *T*. *palmi*, including three inversions and four TDRLs with two sets of alternative scenarios (S6 Fig). The CREx analysis detected inversions of *trnF*, *trnC* and ‘*nad1*-*rrnS*’ gene block, in both scenarios. All the genes in *T*. *palmi* mt genome were encoded by the majority strand except three PCGs (*nad5*, *nad4*, *nad4L*) and three tRNAs (*trnH*, *trnY* and *trnP*). *T*. *palmi* GO showed intense gene rearrangements of 11 PCGs, 22tRNAs, and two rRNAs as compared with the ancestral GO. Most of the genes were transpositioned, while eight genes (*nad1*, *trnL1*, *trnF*, *trnQ*, *trnC*, *trnV*, *rrnS*, and *rrnL*) were inversely transpositioned as compared with the ancestral GO. Further, *T*. *palmi* GO was also compared other thrips mt genomes GO and resulted that *nad2* was conserved between *trnY* and *trnW* in all thrips species. The *cox2* was separated in *T*. *palmi* GO from ‘*cox2*-*trnL2*’ gene block, which was conserved in other thrips species including ancestral GO (Fig 4). The comparison of *T*. *palmi* GO with other thrips mt genomes further revealed *trnD* and *trnR* were translocated and separated from ‘*trnD*-*cox3*’ gene block *in T*. *plami* and *T*. *imaginis*. The gene blocks ‘*cox3*-*nad2*’ and ‘*atp6*-*nad5*’ have been identified as the most frequently rearranged position in thrips mt genomes, while ‘*nad5*-*trnH*-*nad4*-*nad4L*’ seems to be an ancestral gene block. The tRNAs gene blocks ‘*trnQ*-*trnS2*-*trnD*’ and ‘*trnN*-*trnE*-*trnS1*-*trnL1*’ might be specific to genus *Thrips* and *Frankliniella* respectively.

### Strand asymmetry

The strand asymmetry is a remarkable feature of mitochondrial genomes denoted by the AT skew and GC skew on the majority strand (encoded with more genes) [52–54]. Positive AT skew (A>T) and negative GC skew (C>G) is usually observed in most of the insect’s mitochondrial genome with few exceptions; where strand asymmetry is reversed, indicating A<T and C<G on the majority strand [46–47, 50]. A recent study on the mechanism of strand asymmetry in insects confirmed that reversal of strand asymmetry was due to the inversion of replication origin in CRs [54]. We calculated and compared the AT and GC skews of *T*. *palmi* with other thrips mt genomes. The *T*. *palmi* mt genome showed positive AT skew (0.09) and negative GC skew (-0.07) found to be similar with most of the thrips mt genomes. The AT skew of complete mt genomes in other thrips species, ranged from -0.02 (*A*. *obscurus*) to 0.15 (*T*. *imaginis*), while the GC skew varies from -0.11 (*T*. *imaginis*) to 0.01 (*F*. *occidentalis*). The occurrence of Guanine (Gs) is more than Cytosine (Cs) with positive GC skew was observed in *A*. *obscurus* revealed the reversal of strand asymmetry (Table 2). We have also observed and compared the skew value of all PCGs in thrips mt genomes including *T*. *palmi*. The present study depicted that duplication of CRs and structural elements might regulate the extensive gene rearrangements in thrips mt genomes.

### Phylogenetic analysis

**Fig 5:**
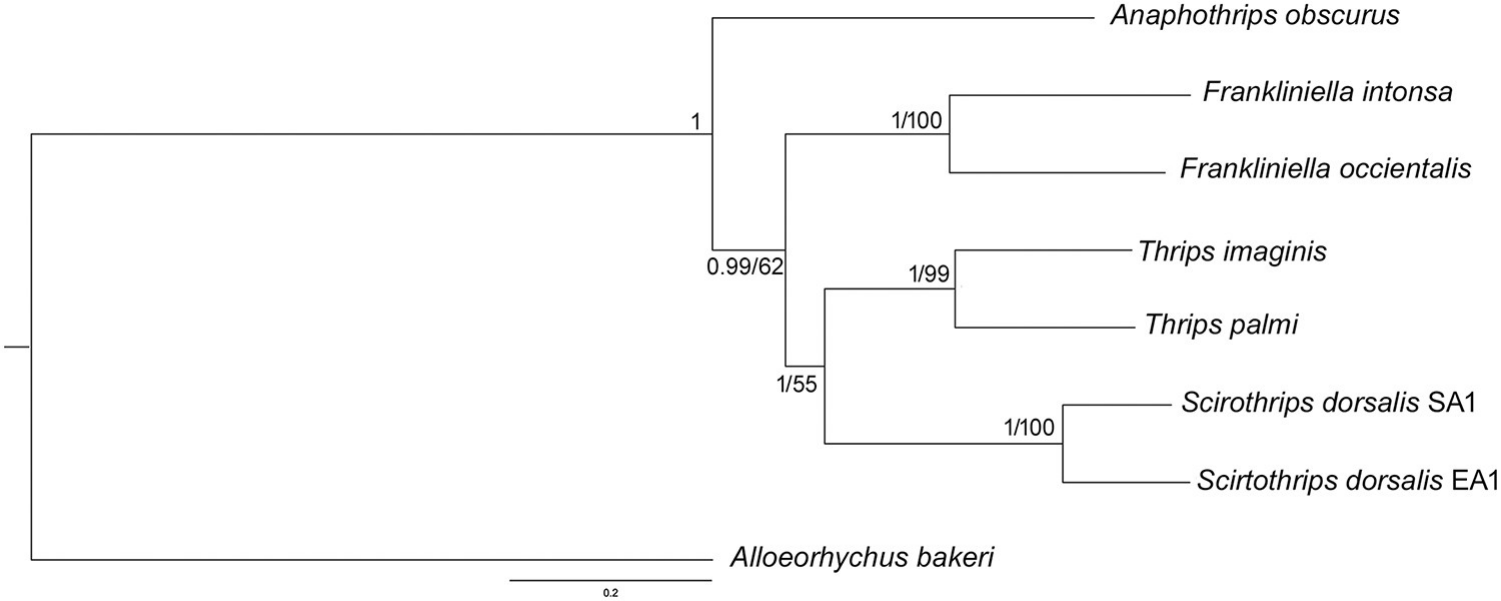
Bayesian phylogenetic tree inferred by 13 PCGs of thrips mt genomes. The Bayesian posterior probabilities and Maximum likelihood bootstrap supports are superimposed with each node. The tree is drawn to scale with values indicated along with the branches (BI/ML).

The Maximum likelihood (ML) and Bayesian Inference (BI) phylogenetic trees were constructed by using 13 PCGs. The *A*. *bakeri* mt genome was used as the out-group in the phylogenetic analysis. The phylogenetic trees generated using both the methods resulted similar topologies (Fig 5). The tree clustering revealed that species under genus *Frankliniella* and *Thrips*, were clustered under the respective genus clade. The phylogenetic analysis showed that genus *Thrips* is more closely related to genus *Scirtothrips* as compared to genus *Frankliniella*. The genus *Thrips* and *Frankliniella* forms two large genus groups ‘*Thrips* genus groups’ and ‘*Frankliniella* genus group’ respectively, which were supposed to be closely related based on the assumed homology for paired ctenidia on abdominal segments V-VIII [55]. However, Mound 2002 suggested that, these two genus-groups are not closely related based on the chaetotaxy of the abdomen and head [55]. Further, the close relationship between *Scirtothrips* and *Thrips* cannot be supported with their morphology. Till date, the comprehensive mt genomes data of thrips species is in its early stages and thus warrant generation of more mt genomes data of diverse thrips species from different hierarchical level to understand the phylogenetic and evolutionary relationships among them.

## Supporting information

S1 Fig Comparative analysis of nucleotide composition of PCGs within the studied thrips species. (TIF)

S2 Fig The Relative Synonymous Codon Usage (RSCU) of the mitochondrial genome of *T*. *palmi* and other thrips species. (TIF)

S3 Fig Comparison of codon usage within the mitochondrial genome of members of the thysanopterans. (TIF)

S4 Fig Transition (S) and transversion (V) saturation plots for PCGs dataset of *T*. *palmi*. (TIF)

S5 Fig Putative secondary structures of the 22 tRNA genes of *T*. *palmi* mt genome. The tRNAs are represented by full names and IUPAC-IUB single letter amino acid codes. The details of stem and loop is mentioned for one tRNA Serine which is applicable for all tRNAs secondary structures.
(TIF)

S6 Fig Evolution of gene order in mitochondrial genome of *T*. *palmi* explained by CREx. In total seven rearrangement operations occurred from the presumed ancestral gene order of *A*. *bakeri* to form the derived gene order of *T*. *palmi* GO. Two alternative sets of scenarios were found, i.e. operations 1–7 and operations 1a–7a.
(TIF)

S1 Table Details of the Thysanoptera mt genomes generated till date and considered for comparative mt genomes study. (DOCX)

S2 Table Start and stop codons of the PCGs of the thrips mt genomes. (DOCX)

S3 Table Nucleotide composition in different mitochondrial locus of the studied thrips mt genomes. (DOCX)

S4 Table RSCU analysis of the PCGs of in *T*. *palmi* mt genome. (DOCX)

S5 Table Genetic distance versus transition-transversion ratio of 13 PCGs in *T*. *palmi* mt genome. (DOCX)

S6 Table Comparison of intergenic nucleotides (INs) and overlapping regions of each gene in thrips mt genomes. (DOCX)

## References

[1] ThripsWiki. ThripsWiki—providing information on the World’s thrips. http://thrips.info/wiki/. (Accessed 1 May 2018).

[2] Tyagi K, Kumar V. Thrips (Insecta: Thysanoptera) of India: An Updated Checklist. Halteres. 2016; 7: 64–98.

[3] Tyagi K, Kumar V. Thrips of Economic importance in India: An identification Guide: 1–96. Published by the Director, Zoological Survey of India, Kolkata 2017; ISBN: 978-81-8171-465-7.

[4] Riley DG, Joseph SV, Srinivasan R, Diffie S. Thrips vectors of tospoviruses. J Int Pest Manage. 2011; 1: 1–10.

[5] Zhou J, Tzanetakis IE. Epidemiology of Soybean vein necrosis-associated virus. Phytopathol. 2013; 103: 966–971, 10.1094/PHYTO-12-12-0322-R 23550970

[6] Buckman RS, Mound LA, Whiting MF. Phylogeny of thrips (Insecta: Thysanoptera) based on five molecular loci. Syst Entomol. 2013; 38: 123–133. 10.1111/j.1365-3113.2012.00650.x

[7] Iftikhar R, Ashfaq M, Rasool A, Hebert PDN. DNA Barcode Analysis of Thrips (Thysanoptera) Diversity in Pakistan Reveals Cryptic Species Complexes. PloS One 2016; 11: e0146014, 10.1371/journal.pone.0146014 26741134 PMC4704811

[8] Tyagi K, Kumar V, Singha D, Chandra K, Laskar BA, Kundu S, et al DNA Barcoding studies on Thrips in India: Cryptic species, Species complexes. Sci Rep. 2017; 7:1–14. 28687754 10.1038/s41598-017-05112-7 PMC5501822

[9] Bhatti JS. Species of the genus Thrips from India (Thysanoptera). Syst Entomol. 1980; 5:109–166.

[10] Singh D, Kabiraj D, Sharma P, Chetia H, Mosahari PV, Neog K, et al The mitochondrial genome of Muga silkworm (Antheraea assamensis) and its comparative analysis with other lepidopteran insects. PloS One. 2017; 12: e0188077, 10.1371/journal.pone.0188077. 29141006 PMC5687760

[11] Hao YJ, Zou YL, Ding YR, Xu WY, Yan ZT, Li XD, et al Complete mitochondrial genomes of *Anopheles stephensi* and *An*. *dirus* and comparative evolutionary mitochondriomics of 50 mosquitoes. Sci Rep. 2017; 7: 7666 10.1038/s41598-017-07977-0 28794438 PMC5550476

[12] Boore JL. Animal mitochondrial genomes. Nucleic Acids Res. 1999; 27: 1767–1780. 10101183 10.1093/nar/27.8.1767 PMC148383

[13] Wolstenholme DR. Animal mitochondrial DNA: structure and evolution. Int Rev Cytol. 1992; 141: 173–216. 1452431 10.1016/s0074-7696(08)62066-5

[14] Shao R, Kirkness EF, Barker SC. The single mitochondrial chromosome typical of animals has evolved into 18 minichromosomes in the human body louse, *Pediculus humanus*. Genome Res. 2009; 19: 904–912. 10.1101/gr.083188.108 19336451 PMC2675979

[15] Shao R, Barker SC. The highly rearranged mitochondrial genome of the plague thrips, *Thrips imaginis* (Insecta: Thysanoptera): convergence of two novel gene boundaries and an extraordinary arrangement of rRNA genes. Mol Biol Evol. 2003; 20: 362–370. 10.1093/molbev/msg045 12644556

[16] Yan D, Tang Y, Xue X, Wang M, Liu F, Fan J. The complete mitochondrial genome sequence of the western flower thrips *Frankliniella occidentalis* (Thysanoptera: Thripidae) contains triplicate putative control regions. Gene. 2012; 506: 117–124. 10.1016/j.gene.2012.06.022 22750320

[17] Yan D, Tang Y, Xue X, Wang M, Liu F, Fan J, et al The mitochondrial genome of *Frankliniella intonsa*: insights into the evolution of mitochondrial genomes at lower taxonomic levels in Thysanoptera. Genomics. 2014; 104: 306–312. 10.1016/j.ygeno.2014.08.003 25128725

[18] Dickey AM, Kumar V, Morgan JK, Cavieres AJ, Shatters RG Jr, McKenzie CL, et al A novel mitochondrial genome architecture in thrips (Insecta: Thysanoptera): extreme size asymmetry among chromosomes and possible recent control region duplication. BMC Genomics. 2015; 16: 439 10.1186/s12864-015-1672-4 26055161 PMC4460840

[19] Liu H, Li H, Song F, Gu W, Feng J, Cai W, et al Novel insights into mitochondrial gene rearrangement in thrips (Insecta: Thysanoptera) from the grass thrips, *Anaphothrips obscurus*. Sci Rep. 2017; 7: 4284 10.1038/s41598-017-04617-5 28655921 PMC5487348

[20] Li H, Durbin R. Fast and accurate short read alignment with Burrows-Wheeler transform. Bioinformatics. 2009; 25: 1754–1760. 10.1093/bioinformatics/btp324 . 19451168 PMC2705234

[21] Bankevich A, Nurk S, Antipov D, Gurevich AA, Dvorkin M, Kulikov AS, et al SPAdes: a new genome assembly algorithm and its applications to single-cell sequencing. J Comput Biol. 2012; 19: 45519:4. 10.1089/cmb.2012.0021 PMC3342519 22506599

[22] Thompson JD, Gibson TJ, Higgins DG. Multiple Sequence Alignment Using ClustalW and ClustalX. Curr Protoc Bioinformatics. 2002; 2.3.1–2.3.22. 10.1002/0471250953.bi0203s00 354. 18792934

[23] Kumar S, Stecher G, Li M, Knyaz C, and Tamura K. MEGA X: Molecular Evolutionary Genetics Analysis across computing platforms. Mol Biol Evol. 2018; 35: 1547–1549. 10.1093/molbev/msy096. 29722887 PMC5967553

[24] Grant JR, Stothard P. The CGView Server: a comparative genomics tool for circular genomes. Nucleic Acids Res. 2008; 36: W181–W184. 10.1093/nar/gkn179 18411202 PMC2447734

[25] Perna NT, Kocher TD. Patterns of nucleotide composition at fourfold degenerate sites of animal mitochondrial genomes. J Mol Evol. 1995; 41: 353–358. 7563121 10.1007/BF00186547

[26] Xia X. DAMBE5: a comprehensive software package for data analysis in molecular biology and evolution. Mol Biol Evol. 2013; 30: 1720–1728. 10.1093/molbev/mst064 23564938 PMC3684854

[27] Abascal F, Zardoya R, Telford MJ. TranslatorX: multiple alignment of nucleotide sequences guided by amino acid translations. Nucleic Acids Res. 2010; 38: W7–W13. 10.1093/nar/gkq291 20435676 PMC2896173

[28] Rozas J, Rozas R. DnaSP, DNA sequence polymorphism: an interactive program for estimating population genetics parameters from DNA sequence data. Comput Appl Biosci. 1995; 11: 621–625. 8808578 10.1093/bioinformatics/11.6.621

[29] Lowe TM, Chan PP. tRNAscan-SE On-line: Search and Contextual Analysis of Transfer RNA Genes. Nucleic Acids Res. 2016; 44: W54–57. 10.1093/nar/gkw413 27174935 PMC4987944

[30] Laslett D. Canbäck B. ARWEN, a program to detect tRNA genes in metazoan mitochondrial nucleotide sequences. Bioinformatics. 2008; 24: 172–175. 10.1093/bioinformatics/btm573 18033792

[31] Reuter JS, Mathews DH. RNAstructure: software for RNA secondary structure prediction and analysis. BMC Bioinformatics. 2010; 11: 129 10.1186/1471-2105-11-129 20230624 PMC2984261

[32] Bernt M, Merkle D, Ramsch K, Fritzsch G, Perseke M, Bernhard D, et al CREx: Inferring Genomic Rearrangements Based on Common Intervals. Bioinformatics. 2007; 23: 2957–2958. 10.1093/bioinformatics/btm468 17895271

[33] Li H, Liu H, Cao L, Shi A, Yang H, Cai W. The complete mitochondrial genome of the damsel bug *Alloeorhynchus bakeri* (Hemiptera: Nabidae). Int J Biol Sci. 2012; 8: 93–107. 22211108 10.7150/ijbs.8.93 PMC3248651

[34] Vaidya G, Lohman DJ, Meier R. SequenceMatrix: concatenation software for the fast assembly of multi-gene datasets with character set and codon information. Cladistics. 2010; 27: 171–180. 10.1111/j.1096-0031.2010.00329.x 34875773

[35] Posada D, Crandall KA. MODELTEST: testing the model of DNA substitution. Bioinformatics. 1998; 14: 817–818. 9918953 10.1093/bioinformatics/14.9.817

[36] Ronquist F, Huelsenbeck JP. MrBayes 3.2: Bayesian phylogenetic inference under mixed models. Bioinformatics. 2003; 19: 1572–1574. 12912839 10.1093/bioinformatics/btg180

[37] Stamatakis A. RAxML Version 8: a tool for Phylogenetic Analysis and Post-Analysis of Large Phylogenies. In Bioinformatics. 2014; 30: 1312–1313. 10.1093/bioinformatics/btu033. 24451623 PMC3998144

[38] Rambaut A. FigTree. Version 1.4.2, Inst Evol Biol Univ. Edinburgh. 2014.

[39] Clary DO, Wolstenholme DR. The mitochondrial DNA molecule of *Drosophila yakuba*: nucleotide sequence, gene organization, and genetic code. J Mol Evol. 1985; 22: 252–271. 10.1007/BF02099755. 3001325

[40] Liu Y, Li Y, Pan M, Dai F, Zhu X, Lu C, et al The complete mitochondrial genome of the Chinese oak silkmoth, *Antheraea pernyi* (Lepidoptera: Saturniidae). Acta Biochim Biophys Sin (Shanghai). 2008; 40: 693–703. 10.1111/j.1745-7270.2008.00449.x. 18685785

[41] Kim SR, Kim MI, Hong MY, Kim KY, Kang PD, Hwang JS, et al The complete mitogenome sequence of the Japanese oak silkmoth, *Antheraea yamamai* (Lepidoptera: Saturniidae). Mol Biol Rep. 2009; 36: 1871–1880. 10.1007/s11033-008-9393-2. 18979227

[42] Shen X, Ren J, Cui Z, Sha Z, Wang B, Xiang J, et al The complete mitochondrial genomes of two common shrimps (*Litopenaeus vannamei* and *Fenneropenaeus chinensis*) and their phylogenomic considerations. Gene. 2007; 403: 98–109. 10.1016/j.gene.2007.06.021. 17890021

[43] Li X, Huang Y, Lei F. Comparative mitochondrial genomics and phylogenetic relationships of the *Crossoptilon* species (Phasianidae, Galliformes). BMC Genomics. 2015; 16: 42 10.1186/s12864-015-1234-9. 25652939 PMC4326528

[44] Meiklejohn CD, Montooth KL, Rand DM. Positive and negative selection on the mitochondrial genome. Trends Genet. 2007; 23: 259–263. 10.1016/j.tig.2007.03.008 17418445

[45] Cameron SL, Johnson KP, Whiting MF. The mitochondrial genome of the screamer louse Bothriometopus (Phthiraptera: Ischnocera): effects of extensive gene rearrangements on the evolution of the genome. J Mol Evol. 2007; 65: 589–604. 10.1007/s00239-007-9042-8 17925995

[46] Hassanin A, Leger N, Deutsch J. Evidence for multiple reversals of asymmetric mutational constraints during the evolution of the mitochondrial genome of Metazoa, and consequences for phylogenetic inferences. Syst Biol. 2005; 54: 277–298. 16021696 10.1080/10635150590947843

[47] Hassanin A. Phylogeny of Arthropoda inferred from mitochondrial sequences: strategies for limiting the misleading effects of multiple changes in pattern and rates of substitution. Mol Phylogenetics Evol. 2006; 38: 100–116. 10.1016/j.ympev.2005.09.012 16290034

[48] Zhang DX, Hewitt GM. Insect mitochondrial control region: a review of its structure, evolution and usefulness in evolutionary studies. Biochem Syst Ecol. 1997; 25: 99–120.

[49] Dowton M, Castro LR, Austin AD. Mitochondrial gene rearrangements as phylogenetic characters in the invertebrates: The examination of genome ‘‘morphology”. Invert Syst. 2002; 16: 345–356.

[50] Cameron SL, Johnson KP, Whiting MF. The mitochondrial genome of the screamer louse *Bothriometopus* (Phthiraptera: Ischnocera): effects of extensive gene rearrangements on the evolution of the genome. J Mol Evol. 2007; 65, 589–604. 10.1007/s00239-007-9042-8 17925995

[51] Chen L, Chen PY, Xue XF, Hua HQ, Li YX, Zhang F, et al Extensive gene rearrangements in the mitochondrial genomes of two egg parasitoids, *Trichogramma japonicum* and *Trichogramma ostriniae* (Hymenoptera: Chalcidoidea: Trichogrammatidae). Sci Rep. 2018; 8: 7034 10.1038/s41598-018-25338-3 29728615 PMC5935716

[52] Nikolaou C, Almirantis Y. Deviations from Chargaff’s second parity rule in organellar DNA—insights into the evolution of organellar genomes. Gene. 2006; 381: 34–41. 10.1016/j.gene.2006.06.010 16893615

[53] Albrecht-Buehler G. Asymptotically increasing compliance of genomes with Chargaff’s second parity rules through inversions and inverted transpositions. Proc Natl Acad Sci USA. 2006; 103: 17828–17833. 10.1073/pnas.0605553103 17093051 PMC1635160

[54] Wei SJ, Shi M, Chen XX, Sharkey MJ, van Achterberg C, Ye GY, et al New Views on Strand Asymmetry in Insect Mitochondrial Genomes. PLoS ONE. 2010; 5: e12708 10.1371/journal.pone.0012708. 20856815 PMC2939890

[55] Mound LA. The Thrips and Frankliniella genus groups: the phylogenetic significance of ctenidia. 2002; Pp. 379–386 in Marullo R & Mound LA [eds] Thrips and Tospoviruses: Proceedings of the 7th International Symposium on Thysanoptera. Australian National Insect Collection, Canberra.

