# Supplementary figures and images for: The complete mitochondrial genome of Melon thrips, Thrips palmi (Thripinae): Comparative analysis
Source: PLoS One. 2018 Oct 31;13(10):e0199404. doi: 10.1371/journal.pone.0199404 (PMC6209132; doi:10.1371/journal.pone.0199404)

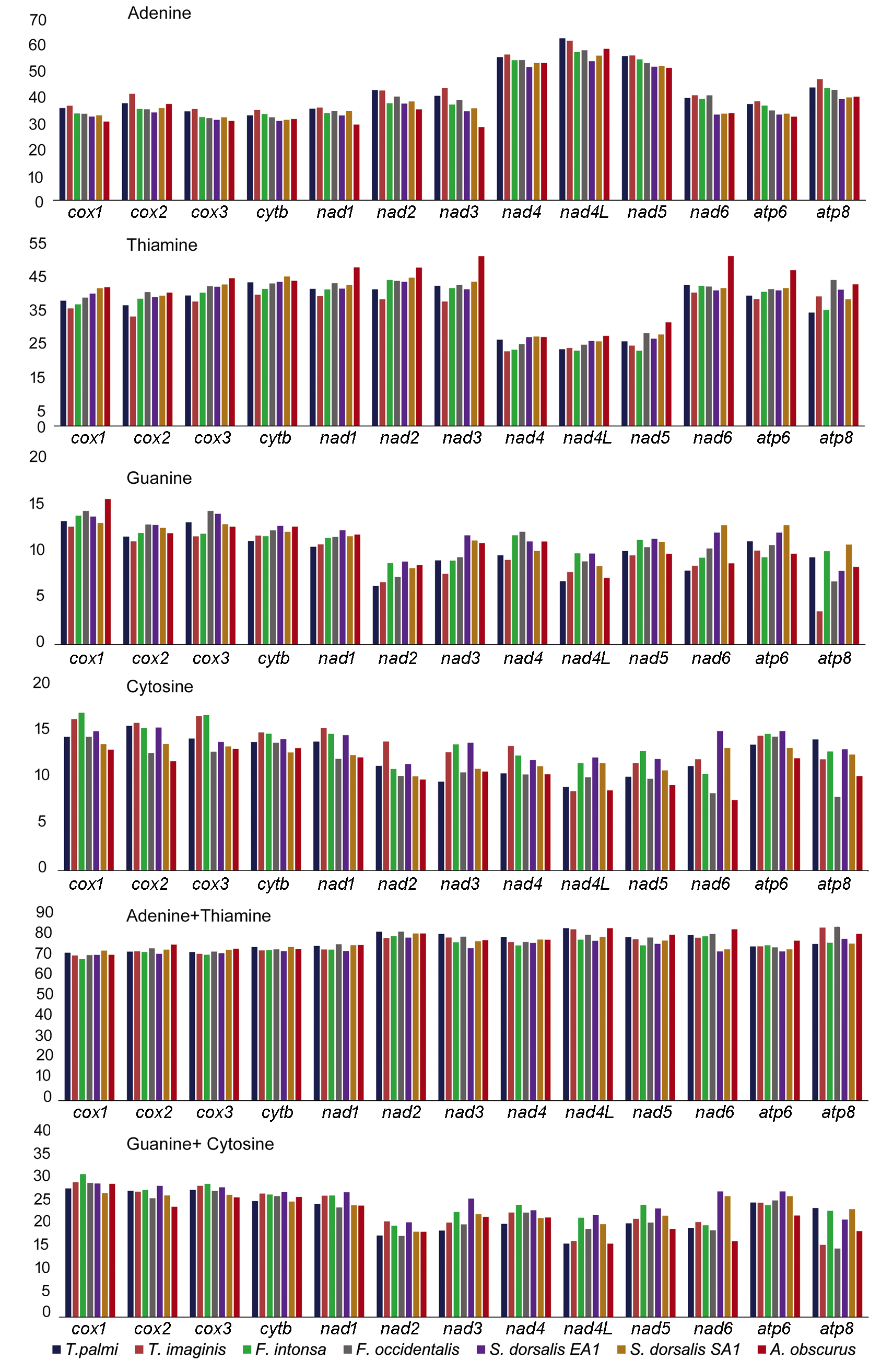

Supplement: S1 Fig — (TIF) [file pone.0199404.s001.tif]

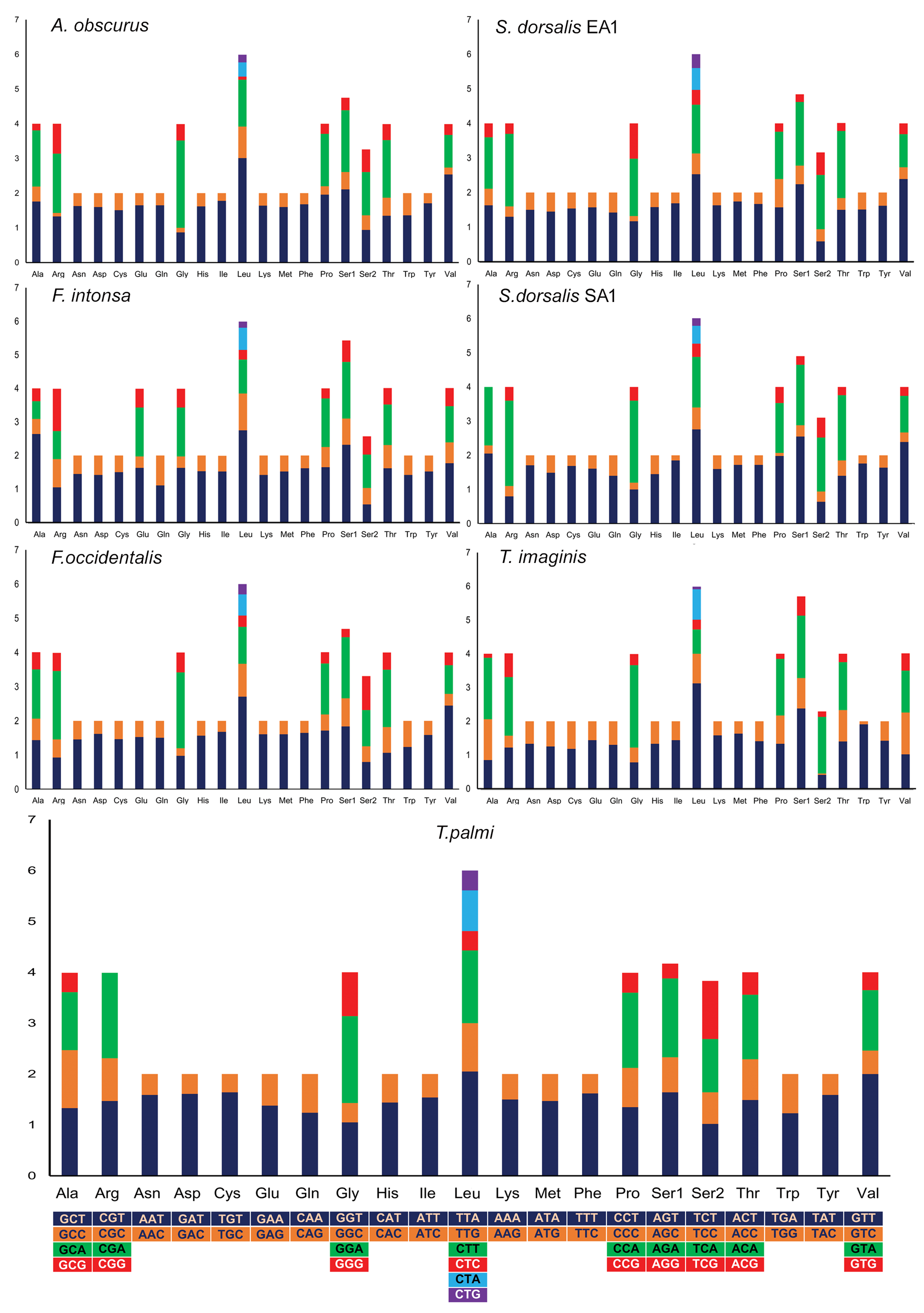

Supplement: S2 Fig — (TIF) [file pone.0199404.s002.tif]

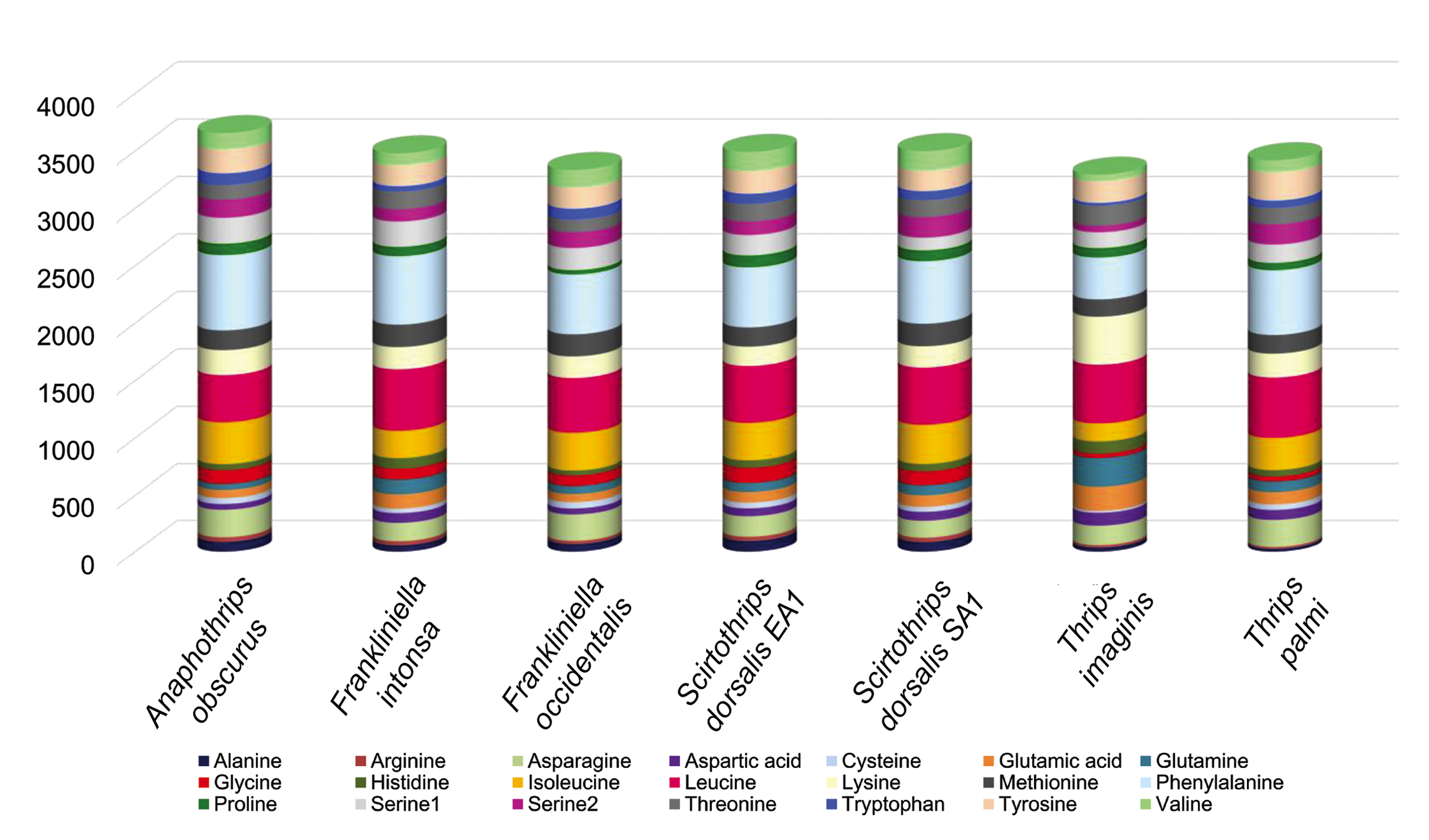

Supplement: S3 Fig — (TIF) [file pone.0199404.s003.tif]

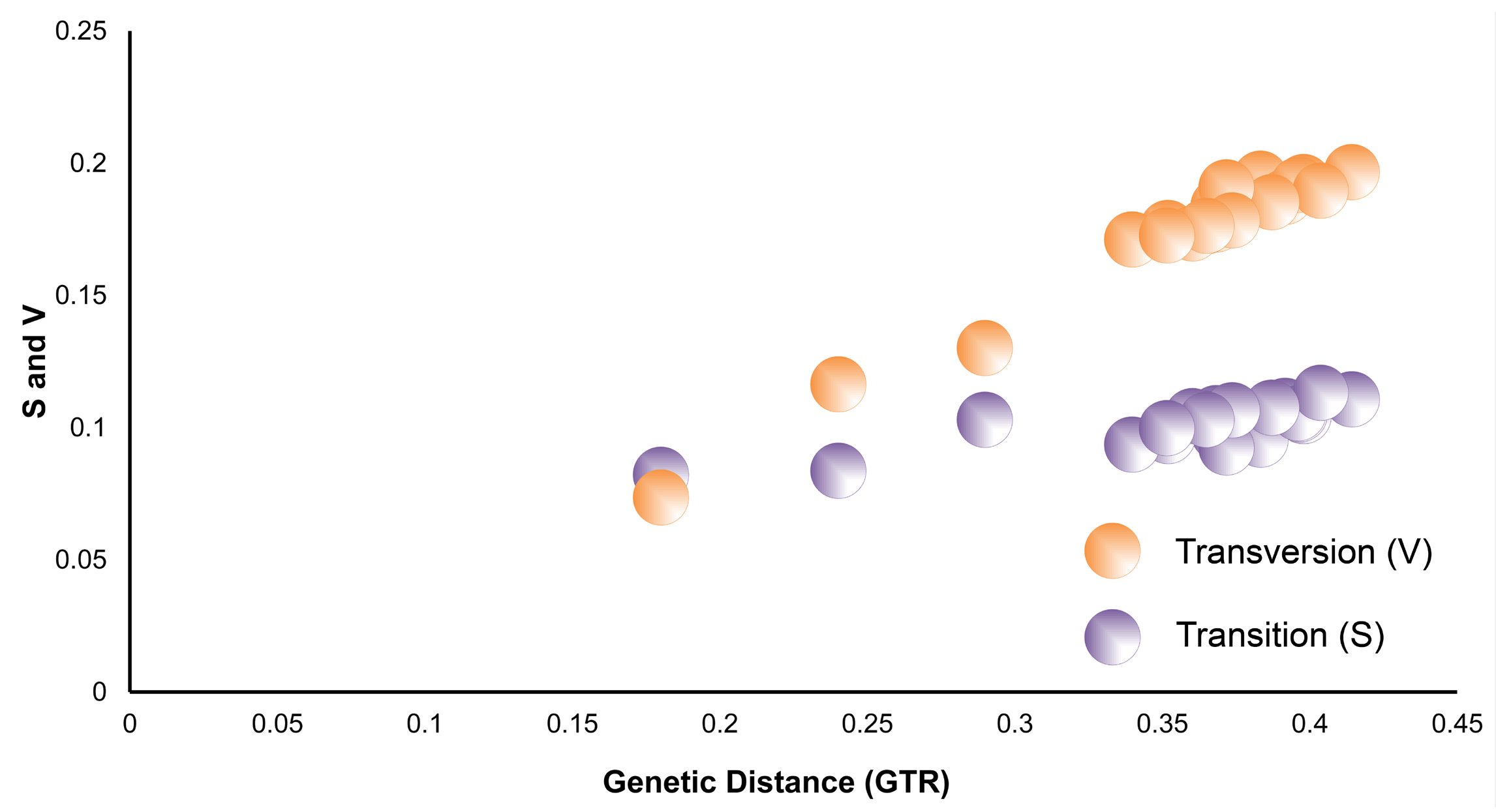

Supplement: S4 Fig — (TIF) [file pone.0199404.s004.tif]

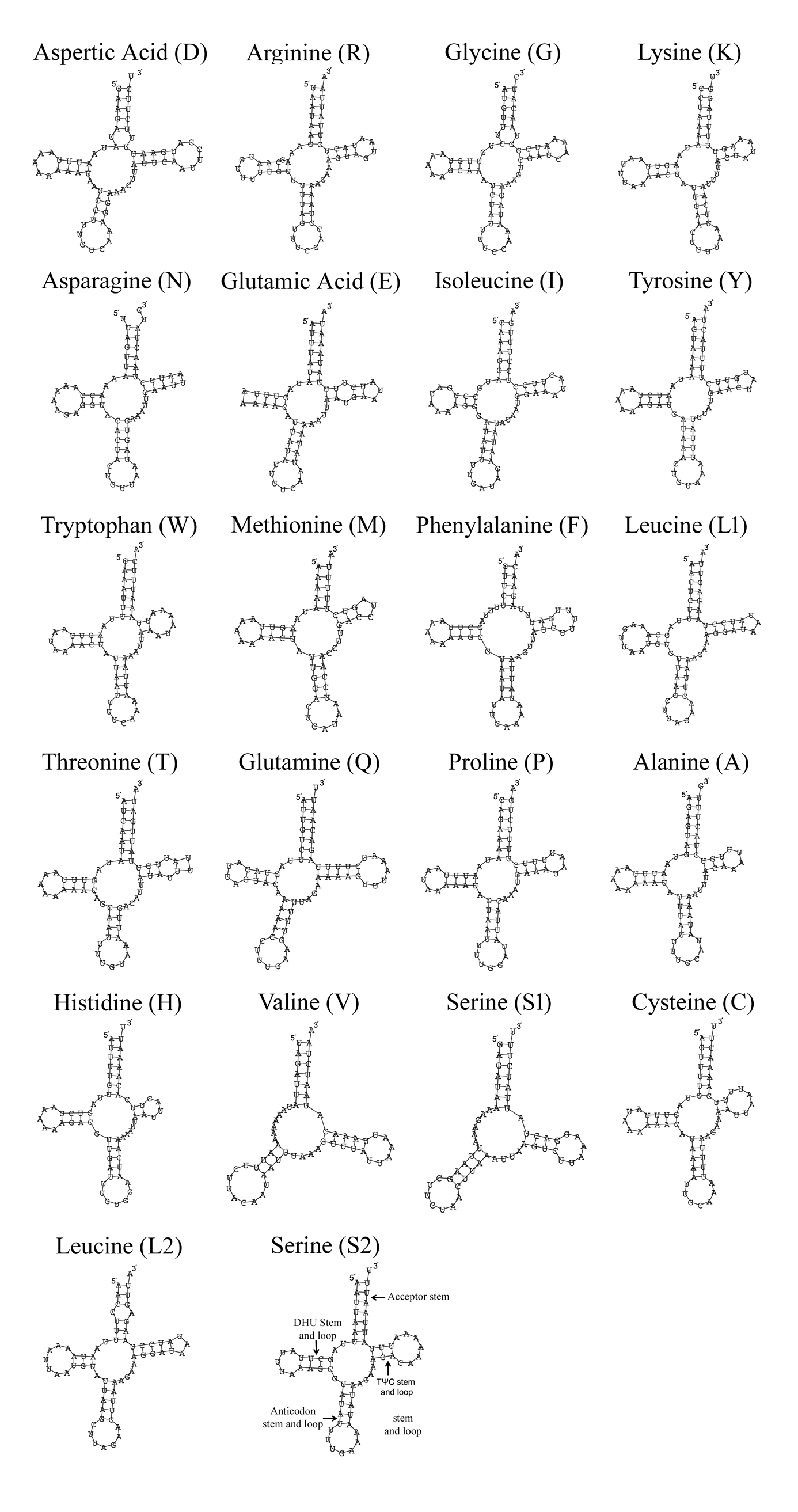

Supplement: S5 Fig — The tRNAs are represented by full names and IUPAC-IUB single letter amino acid codes. The details of stem and loop is mentioned for one tRNA Serine which is applicable for all tRNAs secondary structures. (TIF) [file pone.0199404.s005.tif]

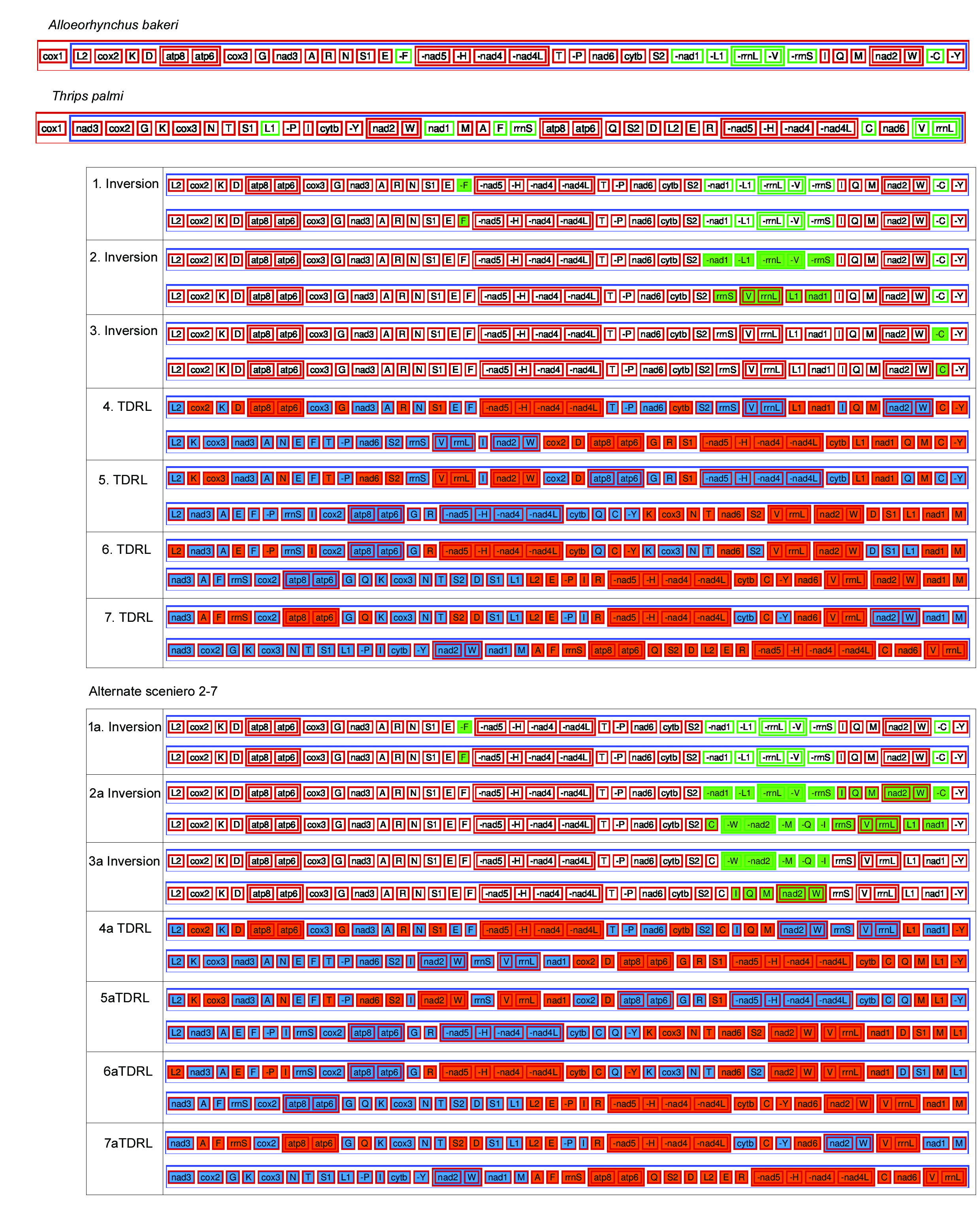

Supplement: S6 Fig — In total seven rearrangement operations occurred from the presumed ancestral gene order of A. bakeri to form the derived gene order of T. palmi GO. Two alternative sets of scenarios were found, i.e. operations 1–7 and operations 1a–7a. (TIF) [file pone.0199404.s006.tif]
