# Supplementary material for: The complete mitochondrial genome of Melon thrips, Thrips palmi (Thripinae): Comparative analysis
Source: PLoS One. 2018 Oct 31;13(10):e0199404. doi: 10.1371/journal.pone.0199404 (PMC6209132; doi:10.1371/journal.pone.0199404)
Supplement: S1 Table — (DOCX) [file pone.0199404.s007.docx]

**S1 Table.**

| **Sub-order** | **Superfamily** | **Family** | **Subfamily** | **Species** | **Accession No.** | **Size (bp)** | **GC%** | **PCG** | **rRNA** | **tRNA** | **CR** | **Reference** |
| --- | --- | --- | --- | --- | --- | --- | --- | --- | --- | --- | --- | --- |
| Terebrantia | Thripoidea | Thripidae | Thripinae | *Thrips palmi* | MH253898 | 15,333 | 22.6 | 13 | 2 | 22 | 2 | This Study |
| Terebrantia | Thripoidea | Thripidae | Thripinae | *Anaphothrips obscurus* | KY498001 | 14,890 | 21.87 | 12 | 2 | 22 | 1 | Liu et al. 2017 |
| Terebrantia | Thripoidea | Thripidae | Thripinae | *Scirtothrips dorsalis* EA1 | KM349826 | 15,343 | 24.26 | 13 | 2 | 22 | 2 | Dickey et al. 2015 |
| Terebrantia | Thripoidea | Thripidae | Thripinae | *Scirtothrips dorsalis* SA1 | KM349827 & KM349828 | 15,204 | 22.6 | 13 | 2 | 22 | 3 | Dickey et al. 2015 |
| Terebrantia | Thripoidea | Thripidae | Thripinae | *Frankliniella intonsa* | JQ917403 | 15,215 | 24.07 | 13 | 2 | 22 | 3 | Yan et al. 2014 |
| Terebrantia | Thripoidea | Thripidae | Thripinae | *Frankliniella occidentalis* | JN835456 | 14,889 | 22.41 | 13 | 2 | 22 | 3 | Yan et al. 2012 |
| Terebrantia | Thripoidea | Thripidae | Thripinae | *Thrips imaginis* | AF335993 | 15,407 | 23.43 | 13 | 2 | 23 | 2 | Shao et al. 2001 |
