# Supplementary material for: The complete mitochondrial genome of Melon thrips, Thrips palmi (Thripinae): Comparative analysis
Source: PLoS One. 2018 Oct 31;13(10):e0199404. doi: 10.1371/journal.pone.0199404 (PMC6209132; doi:10.1371/journal.pone.0199404)
Supplement: S2 Table — (DOCX) [file pone.0199404.s008.docx]

**S2 Table.**

| Gene | *A. obscurus* (KY498001) | | *S. dorsalis* EA1 (KM349826) | | *S. dorsalis* SA1 (KM349827 & KM349828) | | *F. intonsa* (JQ917403) | | *F. occidentalis* (JN835456) | | *T. imaginis* (AF335993) | | *T.palmi*  (MH253898) | |
| --- | --- | --- | --- | --- | --- | --- | --- | --- | --- | --- | --- | --- | --- | --- |
|  | Start | Stop | Start | Stop | Start | Stop | Start | Stop | Start | Stop | Start | Stop | Start | Stop |
| *cox1* | ATC | TAG | ATG | TAA | ATG | TAA | ATG | TAA | ATG | TAA | ATA | TAA | TTG | TAA |
| *cox2* | ATA | TAA | TTG | TAG | TTG | TAG | ATT | TAA | ATA | TAA | ATA | TAA | ATA | TAA |
| *cox3* | ATA | TAA | ATA | TAA | ATA | TAA | ATT | TAA | ATT | TAG | ATA | TAA | ATA | TAA |
| *cytb* | ATA | TAA | ATA | TAG | ATA | TAA | ATA | TAA | ATA | TAA | ATA | TAA | ATA | TAA |
| *atp8* | ATT | T(AA) | ATG | T(AA) | ATG | T(AA) | ATG | T(AA) | ATT | T(AA) | ATA | T(AA) | ATA | T(AA) |
| *atp6* | ATA | TAA | ATT | TAA | ATA | TAA | ATT | TAA | ATT | TAA | ATT | TAA | ATT | TAA |
| *nad1* | ATA | TAA | ATA | TAA | ATA | TAA | ATA | T(AA) | ATT | T(AA) | ATA | T(AA) | ATA | TAA |
| *nad2* | ATA | TAA | ATA | T(AA) | ATA | T(AA) | ATA | T(AA) | ATA | TAA | ATA | T(AA) | ATA | TAA |
| *nad3* | ATA | TAA | ATT | TAA | ATT | TAA | ATA | TAA | ATT | TAA | ATT | TAA | ATG | TAA |
| *nad4* | ATA | TAA | ATT | T(AA) | ATT | T(AA) | ATT | T(AA) | ATT | T(AA) | TAAA | T(AA) | ATT | TAA |
| *nad4L* | ATG | TAA | ATT | T(AA) | ATT | TAA | ATG | T(AA) | ATA | T(AA) | ATT | TAG | ATG | TAG |
| *nad5* | ATA | TAA | ATA | TAG | ATA | TAG | ATT | TAG | ATT | TAA | ATT | T(AA) | ATT | TAA |
| *nad6* | ATA | TAA | ATA | TAA | ATA | TAA | ATA | TAA | ATA | TAA | ATA | TAA | ATT | TAA |
