# Supplementary material for: The complete mitochondrial genome of Melon thrips, Thrips palmi (Thripinae): Comparative analysis
Source: PLoS One. 2018 Oct 31;13(10):e0199404. doi: 10.1371/journal.pone.0199404 (PMC6209132; doi:10.1371/journal.pone.0199404)
Supplement: S3 Table — (DOCX) [file pone.0199404.s009.docx]

**S3 Table.**

|  | **Adenine Composition in different Mitochondrial locus** | | | | | | | | | | | | |
| --- | --- | --- | --- | --- | --- | --- | --- | --- | --- | --- | --- | --- | --- |
| **Species** | ***cox1*** | ***cox2*** | ***cox3*** | ***cytb*** | ***nad1*** | ***nad2*** | ***nad3*** | ***nad4*** | ***nad4L*** | ***nad5*** | ***nad6*** | ***atp6*** | ***atp8*** |
| *T.palmi* | 34.80 | 36.68 | 33.59 | 32.08 | 34.63 | 41.64 | 39.51 | 54.09 | 61.23 | 54.51 | 38.68 | 36.36 | 42.60 |
| *T. imaginis* | 35.73 | 40.21 | 34.48 | 34.14 | 35.04 | 41.52 | 42.45 | 55.09 | 60.30 | 54.75 | 39.69 | 37.44 | 45.78 |
| *F. intonsa* | 32.82 | 34.55 | 31.43 | 32.61 | 32.97 | 36.69 | 36.18 | 52.97 | 56.03 | 53.29 | 38.36 | 35.78 | 42.40 |
| *F.occidentalis* | 32.69 | 34.37 | 31.03 | 31.36 | 33.73 | 39.20 | 37.93 | 52.99 | 56.68 | 51.84 | 39.67 | 33.95 | 41.71 |
| *S. dorsalis EA1* | 31.64 | 33.19 | 30.42 | 30.00 | 32.04 | 36.54 | 33.62 | 50.38 | 52.57 | 50.44 | 32.40 | 32.40 | 38.29 |
| *S.dorsalis* SA1 | 32.10 | 34.81 | 31.35 | 30.45 | 33.76 | 37.35 | 34.75 | 51.90 | 54.65 | 50.77 | 32.72 | 32.72 | 38.86 |
| *A.obscurus* | 29.76 | 36.39 | 30.04 | 30.70 | 28.60 | 34.35 | 27.68 | 51.90 | 57.25 | 50.03 | 32.94 | 31.59 | 39.16 |
|  | **Thiamine Composition in different Mitochondrial locus** | | | | | | | | | | | | |
| **Species** | ***cox1*** | ***cox2*** | ***cox3*** | ***cytb*** | ***nad1*** | ***nad2*** | ***nad3*** | ***nad4*** | ***nad4L*** | ***nad5*** | ***nad6*** | ***atp6*** | ***atp8*** |
| *T.palmi* | 37.30 | 35.92 | 38.83 | 42.77 | 40.80 | 40.66 | 41.73 | 25.68 | 22.83 | 25.17 | 41.98 | 38.79 | 33.73 |
| *T. imaginis* | 35.00 | 32.59 | 37.04 | 39.07 | 38.63 | 37.72 | 37.04 | 22.23 | 23.22 | 23.92 | 39.69 | 37.75 | 38.55 |
| *F. intonsa* | 36.20 | 37.88 | 39.67 | 40.79 | 40.65 | 43.47 | 41.03 | 22.70 | 22.41 | 22.40 | 41.72 | 39.94 | 34.56 |
| *F. occidentalis* | 38.20 | 39.85 | 41.60 | 42.41 | 42.49 | 43.18 | 41.95 | 24.35 | 24.19 | 27.64 | 41.51 | 40.74 | 43.43 |
| *S. dorsalis EA1* | 39.40 | 38.35 | 41.44 | 42.88 | 40.88 | 42.91 | 40.68 | 26.45 | 25.30 | 25.99 | 40.34 | 40.34 | 40.57 |
| *S.dorsalis* SA1 | 41.00 | 38.79 | 42.13 | 44.50 | 41.96 | 44.13 | 42.94 | 26.63 | 25.19 | 27.22 | 41.05 | 41.05 | 37.71 |
| *A.obscurus* | 41.30 | 39.68 | 43.98 | 43.23 | 47.20 | 47.15 | 50.56 | 26.48 | 26.81 | 30.86 | 50.59 | 46.38 | 42.17 |
|  | **Guanine Composition in different Mitochondrial locus** | | | | | | | | | | | | |
| **Species** | ***cox1*** | ***cox2*** | ***cox3*** | ***cytb*** | ***nad1*** | ***nad2*** | ***nad3*** | ***nad4*** | ***nad4L*** | ***nad5*** | ***nad6*** | ***atp6*** | ***atp8*** |
| *T.palmi* | 13.40 | 11.72 | 13.28 | 11.23 | 10.61 | 6.35 | 9.14 | 9.70 | 6.88 | 10.16 | 8.02 | 11.21 | 9.47 |
| *T. imaginis* | 12.80 | 11.21 | 11.75 | 11.83 | 10.88 | 6.78 | 7.69 | 9.19 | 7.87 | 9.69 | 8.55 | 10.20 | 3.61 |
| *F. intonsa* | 14.00 | 12.12 | 12.04 | 11.77 | 11.57 | 8.84 | 9.12 | 11.87 | 9.91 | 11.35 | 9.43 | 9.48 | 10.14 |
| *F. occidentalis* | 14.50 | 13.04 | 14.50 | 12.40 | 11.68 | 7.36 | 9.48 | 12.25 | 9.03 | 10.57 | 10.43 | 10.80 | 6.86 |
| *S. dorsalis EA1* | 13.90 | 12.98 | 14.20 | 12.88 | 12.41 | 9.01 | 11.86 | 11.20 | 9.88 | 11.49 | 12.15 | 12.15 | 8.00 |
| *S. dorsalis SA1* | 13.20 | 12.68 | 13.07 | 12.25 | 11.76 | 8.30 | 11.30 | 10.17 | 8.53 | 11.15 | 12.96 | 12.96 | 10.86 |
| *A. obscurus* | 15.80 | 12.09 | 12.80 | 12.81 | 11.94 | 8.64 | 11.02 | 11.19 | 7.25 | 9.85 | 8.82 | 9.86 | 8.43 |
|  | **Cytosine Composition in different Mitochondrial locus** | | | | | | | | | | | | |
| **Species** | ***cox1*** | ***cox2*** | ***cox3*** | ***cytb*** | ***nad1*** | ***nad2*** | ***nad3*** | ***nad4*** | ***nad4L*** | ***nad5*** | ***nad6*** | ***atp6*** | ***atp8*** |
| *T.palmi* | 14.50 | 15.68 | 14.30 | 13.93 | 13.96 | 11.34 | 9.63 | 10.53 | 9.06 | 10.16 | 11.32 | 13.64 | 14.20 |
| *T. imaginis* | 16.40 | 15.99 | 16.73 | 14.96 | 15.45 | 13.98 | 12.82 | 13.49 | 8.61 | 11.64 | 12.06 | 14.61 | 12.05 |
| *F. intonsa* | 17.10 | 15.45 | 16.86 | 14.82 | 14.81 | 11.00 | 13.68 | 12.46 | 11.64 | 12.96 | 10.48 | 14.80 | 12.90 |
| *F. occidentalis* | 14.50 | 12.74 | 12.87 | 13.84 | 12.11 | 10.25 | 10.63 | 10.41 | 10.11 | 9.94 | 8.38 | 14.51 | 8.00 |
| *S. dorsalis EA1* | 15.10 | 15.49 | 13.94 | 14.23 | 14.67 | 11.54 | 13.84 | 11.97 | 12.25 | 12.08 | 15.11 | 15.11 | 13.14 |
| *S. dorsalis SA1* | 13.70 | 13.72 | 13.45 | 12.79 | 12.51 | 10.22 | 11.02 | 11.31 | 11.63 | 10.85 | 13.27 | 13.27 | 12.57 |
| *A. obscurus* | 13.10 | 11.84 | 13.18 | 13.26 | 12.26 | 9.86 | 10.73 | 10.43 | 8.70 | 9.26 | 7.65 | 12.17 | 10.24 |
|  | **Adenine + Thiamine Composition in different Mitochondrial locus** | | | | | | | | | | | | |
| **Species** | ***cox1*** | ***cox2*** | ***cox3*** | ***cytb*** | ***nad1*** | ***nad2*** | ***nad3*** | ***nad4*** | ***nad4L*** | ***nad5*** | ***nad6*** | ***atp6*** | ***atp8*** |
| *T.palmi* | 72.10 | 72.60 | 72.41 | 74.84 | 75.43 | 82.31 | 81.23 | 79.77 | 84.06 | 79.67 | 80.66 | 75.15 | 76.33 |
| *T. imaginis* | 70.74 | 72.80 | 71.52 | 73.21 | 73.67 | 79.24 | 79.49 | 77.32 | 83.52 | 78.68 | 79.39 | 75.19 | 84.34 |
| *F. intonsa* | 68.98 | 72.43 | 71.10 | 73.41 | 73.62 | 80.16 | 77.21 | 75.67 | 78.45 | 75.69 | 80.08 | 75.72 | 76.96 |
| *F. occidentalis* | 70.91 | 74.22 | 72.63 | 73.76 | 76.22 | 82.39 | 79.89 | 77.34 | 80.87 | 79.49 | 81.19 | 74.69 | 85.14 |
| *S. dorsalis EA1* | 71.02 | 71.54 | 71.86 | 72.88 | 72.92 | 79.45 | 74.29 | 76.83 | 77.87 | 76.44 | 72.74 | 72.74 | 78.86 |
| *S. dorsalis SA1* | 73.10 | 73.60 | 73.48 | 74.95 | 75.73 | 81.48 | 77.68 | 78.53 | 79.84 | 78.00 | 73.77 | 73.77 | 76.57 |
| *A. obscurus* | 71.09 | 76.07 | 74.02 | 73.93 | 75.81 | 81.50 | 78.25 | 78.39 | 84.06 | 80.88 | 83.53 | 77.97 | 81.33 |
|  | **Guanine + Cytosine Composition in different Mitochondrial locus** | | | | | | | | | | | | |
| **Species** | ***cox1*** | ***cox2*** | ***cox3*** | ***cytb*** | ***nad1*** | ***nad2*** | ***nad3*** | ***nad4*** | ***nad4L*** | ***nad5*** | ***nad6*** | ***atp6*** | ***atp8*** |
| *T.palmi* | 27.90 | 27.40 | 27.59 | 25.16 | 24.57 | 17.69 | 18.77 | 20.23 | 15.94 | 20.33 | 19.34 | 24.85 | 23.67 |
| *T. imaginis* | 29.26 | 27.20 | 28.48 | 26.79 | 26.33 | 20.76 | 20.51 | 22.68 | 16.48 | 21.32 | 20.61 | 24.81 | 15.66 |
| *F. intonsa* | 31.02 | 27.57 | 28.90 | 26.59 | 26.38 | 19.84 | 22.79 | 24.33 | 21.55 | 24.31 | 19.92 | 24.28 | 23.04 |
| *F. occidentalis* | 29.09 | 25.78 | 27.37 | 26.24 | 23.78 | 17.61 | 20.11 | 22.66 | 19.13 | 20.51 | 18.81 | 25.31 | 14.86 |
| *S. dorsalis EA1* | 28.98 | 28.47 | 28.14 | 27.12 | 27.08 | 20.55 | 25.71 | 23.17 | 22.13 | 23.56 | 27.26 | 27.26 | 21.14 |
| *S. dorsalis SA1* | 26.90 | 26.40 | 26.52 | 25.05 | 24.27 | 18.52 | 22.32 | 21.47 | 20.16 | 22.00 | 26.23 | 26.23 | 23.43 |
| *A. obscurus* | 28.91 | 23.93 | 25.98 | 26.07 | 24.19 | 18.50 | 21.75 | 21.61 | 15.94 | 19.12 | 16.47 | 22.03 | 18.67 |
