# Supplementary material for: The complete mitochondrial genome of Melon thrips, Thrips palmi (Thripinae): Comparative analysis
Source: PLoS One. 2018 Oct 31;13(10):e0199404. doi: 10.1371/journal.pone.0199404 (PMC6209132; doi:10.1371/journal.pone.0199404)
Supplement: S4 Table — (DOCX) [file pone.0199404.s010.docx]

**S4 Table.**

| **Amino acid** | **Codon** | **Number** | **Frequency (%)** | **RSCU** | **Amino acid** | **Codon** | **Number** | **Frequency (%)** | **RSCU** |
| --- | --- | --- | --- | --- | --- | --- | --- | --- | --- |
| Phenylalanine | TTT | 458 | 12.46 | 1.62 | Tyrosine | TAT | 197 | 5.36 | 1.59 |
|  | TTC | 107 | 2.91 | 0.38 |  | TAC | 51 | 1.39 | 0.41 |
| Leucine | TTA | 179 | 4.87 | 2.05 | **Stop codon** | TAA* | 187 | 5.09 | 1.44 |
|  | TTG | 83 | 2.26 | 0.95 |  | TAG* | 72 | 1.96 | 0.56 |
|  | CTT | 125 | 3.40 | 1.43 | Histidine | CAT | 41 | 1.12 | 1.44 |
|  | CTC | 33 | 0.90 | 0.38 |  | CAC | 16 | 0.44 | 0.56 |
|  | CTA | 70 | 1.90 | 0.8 | Glutamine | CAA | 57 | 1.55 | 1.24 |
|  | CTG | 34 | 0.92 | 0.39 |  | CAG | 35 | 0.95 | 0.76 |
| Isoleucine | ATT | 217 | 5.90 | 1.54 | Asparagine | AAT | 191 | 5.20 | 1.59 |
|  | ATC | 64 | 1.74 | 0.46 |  | AAC | 50 | 1.36 | 0.41 |
| Methionine | ATA | 119 | 3.24 | 1.47 | Lysine | AAA | 157 | 4.27 | 1.5 |
|  | ATG | 43 | 1.17 | 0.53 |  | AAG | 52 | 1.41 | 0.5 |
| Valine | GTT | 52 | 1.41 | 2 | Aspartic acid | GAT | 71 | 1.93 | 1.61 |
|  | GTC | 12 | 0.33 | 0.46 |  | GAC | 17 | 0.46 | 0.39 |
|  | GTA | 31 | 0.84 | 1.19 | Glutamic acid | GAA | 75 | 2.04 | 1.38 |
|  | GTG | 9 | 0.24 | 0.35 |  | GAG | 34 | 0.92 | 0.62 |
| Serine | TCT | 69 | 1.88 | 1.64 | Cysteine | TGT | 41 | 1.12 | 1.64 |
|  | TCC | 29 | 0.79 | 0.69 |  | TGC | 9 | 0.24 | 0.36 |
|  | TCA | 65 | 1.77 | 1.55 | Tryptophan | TGA | 38 | 1.03 | 1.23 |
|  | TCG | 12 | 0.33 | 0.29 |  | TGG | 24 | 0.65 | 0.77 |
| Proline | CCT | 21 | 0.57 | 1.35 | Arginine | CGT | 7 | 0.19 | 1.47 |
|  | CCC | 12 | 0.33 | 0.77 |  | CGC | 4 | 0.11 | 0.84 |
|  | CCA | 23 | 0.63 | 1.48 |  | CGA | 8 | 0.22 | 1.68 |
|  | CCG | 6 | 0.16 | 0.39 |  | CGG | 0 | 0.00 | 0 |
|  | ACT | 54 | 1.47 | 1.49 | Serine | AGT | 43 | 1.17 | 1.02 |
|  | ACC | 29 | 0.79 | 0.8 |  | AGC | 26 | 0.71 | 0.62 |
|  | ACA | 46 | 1.25 | 1.27 |  | AGA | 44 | 1.20 | 1.05 |
|  | ACG | 16 | 0.44 | 0.44 |  | AGG | 48 | 1.31 | 1.14 |
| Alanine | GCT | 7 | 0.19 | 1.33 | Glycine | GGT | 11 | 0.30 | 1.05 |
|  | GCC | 6 | 0.16 | 1.14 |  | GGC | 4 | 0.11 | 0.38 |
|  | GCA | 6 | 0.16 | 1.14 |  | GGA | 18 | 0.49 | 1.71 |
|  | GCG | 2 | 0.05 | 0.38 |  | GGG | 9 | 0.24 | 0.86 |
