# Supplementary material for: The complete mitochondrial genome of Melon thrips, Thrips palmi (Thripinae): Comparative analysis
Source: PLoS One. 2018 Oct 31;13(10):e0199404. doi: 10.1371/journal.pone.0199404 (PMC6209132; doi:10.1371/journal.pone.0199404)
Supplement: S5 Table — (DOCX) [file pone.0199404.s011.docx]

**S5 Table.**

| **Genetic distance** | **Transition (S)** | **Transversion (V)** |
| --- | --- | --- |
| 0.414271 | 0.110767 | 0.196679 |
| 0.397912 | 0.104373 | 0.192966 |
| 0.289842 | 0.103032 | 0.130157 |
| 0.396574 | 0.105095 | 0.191832 |
| 0.368219 | 0.105507 | 0.176877 |
| 0.364598 | 0.103548 | 0.176052 |
| 0.383308 | 0.095513 | 0.194224 |
| 0.391659 | 0.108303 | 0.187313 |
| 0.36908 | 0.098092 | 0.183909 |
| 0.351927 | 0.096854 | 0.175554 |
| 0.396149 | 0.105507 | 0.191832 |
| 0.403699 | 0.113243 | 0.189666 |
| 0.387092 | 0.10757 | 0.185334 |
| 0.360244 | 0.104373 | 0.173474 |
| 0.179998 | 0.082207 | 0.073646 |
| 0.371778 | 0.092512 | 0.190904 |
| 0.373707 | 0.106642 | 0.178424 |
| 0.365013 | 0.103032 | 0.176361 |
| 0.240163 | 0.083746 | 0.11644 |
| 0.339803 | 0.093657 | 0.171222 |
| 0.351555 | 0.099835 | 0.172752 |
