# Supplementary material for: The complete mitochondrial genome of Melon thrips, Thrips palmi (Thripinae): Comparative analysis
Source: PLoS One. 2018 Oct 31;13(10):e0199404. doi: 10.1371/journal.pone.0199404 (PMC6209132; doi:10.1371/journal.pone.0199404)
Supplement: S6 Table — (DOCX) [file pone.0199404.s012.docx]

**S6 Table.**

| ***T. palmi*** | **INs** | ***T.imaginis*** | **INs** | ***F. intonsa*** | **INs** | ***F. occidentalis*** | **INs** | ***S. dorsalis* EA1** | **INs** | ***S. dorsalis* SA1** | **INs** | ***A. obscurus*** | **INs** |
| --- | --- | --- | --- | --- | --- | --- | --- | --- | --- | --- | --- | --- | --- |
| *nad5* | 29 | *nad5* | -3 | *Q* | 0 | *cox3* | 0 | *cox1* | 0 | *cox1* | -7 | *cox1* | -1 |
| *H* | 2 | *H* | 0 | CR3 | 0 | CR2 | 0 | *nad3* | 0 | *nad3* | -1 | *L2* | 1 |
| *nad4* | -7 | *nad4* | -21 | *P* | -1 | *T* | 6 | *L2* | 0 | *L2* | 0 | *cox2* | 18 |
| *nad4L* | 34 | *nad4L* | 40 | *Y* | 33 | *Q* | 49 | *cox2* | -2 | *cox2* | -2 | *D* | -1 |
| *C* | 19 | *C* | 49 | *nad2* | 0 | *I* | 1 | *D* | -1 | *D* | -1 | *R* | -3 |
| *nad6* | 43 | *nad6* | 39 | *W* | 0 | *cytb* | 0 | *R* | 0 | *R* | 42 | *G* | 4 |
| *V* | -31 | *V* | 0 | *nad1* | 0 | CR3 | 0 | CR2 | 0 | *G* | -1 | *K* | 7 |
| *rrnL* | 27 | *rrnL* | 0 | *M* | 1 | *P* | -1 | *G* | -1 | *K* | 11 | *cox3* | 3 |
| *cox1* | -1 | *cox1* | 2 | *A* | 0 | *Y* | 4 | *K* | 14 | *cox3* | 0 | *nad3* | 18 |
| *nad3* | 12 | *nad3* | -1 | *F* | 0 | *nad2* | 0 | *cox3* | 1 | *I* | 0 | *N* | -5 |
| *cox2* | 5 | *L2* | 0 | *rrnS* | 0 | *W* | 1 | *I* | 0 | *L1* | 0 | *E* | 0 |
| *G* | -1 | *cox2* | 2 | *atp8* | -2 | *nad1* | 0 | *L1* | 0 | *T* | 3 | *Q* | -4 |
| *K* | 13 | *G* | 0 | *atp6* | 8 | *M* | 3 | *T* | 3 | *P* | 16 | *I* | 4 |
| *cox3* | 4 | *K* | 11 | *N* | -2 | *A* | -1 | *P* | 0 | *N* | -4 | *cytb* | 2 |
| *N* | -3 | *cox3* | 13 | *E* | -1 | *F* | 0 | *N* | -3 | *E* | 1 | *Y* | 31 |
| *T* | 7 | *R* | 11 | *S1* | -1 | *rrnS* | 0 | *E* | 1 | *Q* | 45 | *nad2* | 0 |
| *S1* | 15 | *T* | 0 | *L1* | 0 | *atp8* | 5 | *Q* | 45 | *cytb* | 11 | *W* | 0 |
| *L1* | 3 | *N* | -3 | CR1 | 0 | *atp6* | 3 | *cytb* | 4 | *Y* | 25 | *nad1* | 29 |
| CR2 | 1 | *E* | 0 | nad5 | 0 | *N* | 0 | *Y* | 29 | *nad2* | -1 | *A* | -4 |
| *P* | 23 | CR2 | 0 | *H* | 0 | *E* | 0 | *nad2* | -1 | *W* | -1 | *F* | 0 |
| *I* | 1 | *S3* | 1 | *nad4* | -2 | *S1* | 0 | *W* | -1 | *nad1* | -4 | *rrnS* | 0 |
| *cytb* | 8 | *P* | 3 | *nad4L* | 19 | *L1* | -2 | *nad1* | -4 | *M* | 0 | *T* | 8 |
| *Y* | 37 | *I* | 1 | *C* | 17 | CR1 | 0 | *M* | 0 | *A* | -4 | *M* | 36 |
| *nad2* | -50 | *cytb* | 0 | *nad6* | 32 | *nad5* | 77 | *A* | -3 | *F* | -1 | *atp8* | -10 |
| *W* | 0 | *Y* | 23 | *V* | 0 | *H* | 0 | *F* | -2 | *rrnS* | 85 | *atp6* | 27 |
| *nad1* | -4 | *nad2* | 0 | *rrnL* | 0 | *nad4* | -2 | *rrnS* | 85 | *atp8* | 17 | *S1* | 0 |
| *M* | 1 | *W* | 0 | *S2* | 1 | *nad4L* | 17 | *atp8* | 23 | *atp6* | -2 | CR | 0 |
| *A* | -1 | *nad1* | 0 | *cox1* | 2 | *C* | 11 | *atp6* | -2 | *S1* | 0 | *nad5* | -2 |
| *F* | -2 | *M* | 1 | *nad3* | 1 | *nad6* | 34 | *S1* | 0 | CR1 | 0 | *H* | 10 |
| *rrnS* | -1 | *A* | -1 | *L2* | 0 | *V* | 0 | CR1 | 0 | *nad5* | 0 | *nad4* | 0 |
| *atp8* | -7 | *F* | 0 | *cox2* | 3 | *rrnL* | -66 | *nad5* | -3 | CR2 | 0 | *nad4L* | 0 |
| *atp6* | -1 | *rrnS* | 0 | *D* | 0 | *S2* | 1 | *H* | 150 | *H* | -1 | *L1* | -1 |
| *Q* | 41 | *atp8* | -4 | *R* | 3 | *cox1* | 5 | *nad4* | 5 | *nad4* | -7 | *C* | 4 |
| *S2* | 1 | *atp6* | 1 | *G* | -1 | *nad3* | -1 | *nad4L* | 47 | *nad4L* | 74 | *nad6* | 3 |
| *D* | 46 | *Q* | -2 | *K* | 17 | *L2* | 0 | *C* | 13 | *V* | 1 | *P* | 85 |
| *L2* | 99 | *S2* | 1 | *cox3* | 17 | *cox2* | 0 | *nad6* | 19 | *rrnL* | -49 | *V* | 15 |
| *E* | 49 | *D* | 11 | *I* | 18 | *D* | 0 | *V* | 0 | *S2* | 0 | *rrnL* | 4 |
| *R* | 0 | *S1* | 0 | *T* | 0 | *R* | -1 | *rrnL* | -49 | *C* | 11 | *S2* | 0 |
| CR1 | 0 | *L1* | 8 | CR2 | 0 | *G* | -1 | *S2* | - | *nad6* | 0 | - | - |
| - | - | *E* | 0 | *cytb* | - | *K* | - | - | - | CR3 | - | - | - |
| - | - | CR1 | - | - | - | - | - | - | - | - | - | - | - |
